# Characterization of Novel Derivatives of MBQ-167, an Inhibitor of the GTP-binding Proteins Rac/Cdc42

**DOI:** 10.1158/2767-9764.CRC-22-0303

**Published:** 2022-12-29

**Authors:** Julia I. Medina, Ailed Cruz-Collazo, Maria del Mar Maldonado, Tatiana Matos Gascot, Luis D. Borrero-Garcia, Mariana Cooke, Marcelo G. Kazanietz, Eliud Hernandez O'Farril, Cornelis P. Vlaar, Suranganie Dharmawardhane

**Affiliations:** 1 Department of Biochemistry, School of Medicine, University of Puerto Rico, San Juan, Puerto Rico.; 2 Department of Pharmaceutical Sciences, School of Pharmacy, University of Puerto Rico, San Juan, Puerto Rico.; 3 MBQ Pharma, Inc., San Juan, Puerto Rico.; 4 Department of Systems Pharmacology and Translational Therapeutics, Perelman School of Medicine, University of Pennsylvania, Philadelphia, Pennsylvania.

## Abstract

Rac and Cdc42, are homologous GTPases that regulate cell migration, invasion, and cell-cycle progression; thus, representing key targets for metastasis therapy. We previously reported on the efficacy of MBQ-167, which blocks both Rac1 and Cdc42 in breast cancer cells and mouse models of metastasis. To identify compounds with increased activity, a panel of MBQ-167 derivatives was synthesized, maintaining its 9-ethyl-3-(1*H*-1,2,3-triazol-1-yl)-9*H*-carbazole core. Similar to MBQ-167, MBQ-168 and EHop-097 inhibit activation of Rac and Rac1B splice variant and breast cancer cell viability, and induce apoptosis. MBQ-167 and MBQ-168 inhibit Rac and Cdc42 by interfering with guanine nucleotide binding, and MBQ-168 is a more effective inhibitor of P21-activated kinase (1–3) activation. EHop-097 acts via a different mechanism by inhibiting the interaction of the guanine nucleotide exchange factor Vav with Rac. MBQ-168 and EHop-097 inhibit metastatic breast cancer cell migration, and MBQ-168 promotes loss of cancer cell polarity to result in disorganization of the actin cytoskeleton and detachment from the substratum. In lung cancer cells, MBQ-168 is more effective than MBQ-167 or EHop-097 at reducing ruffle formation in response to EGF. Comparable with MBQ-167, MBQ-168 significantly inhibits HER2-positive tumor growth and metastasis to lung, liver, and spleen. Both MBQ-167 and MBQ-168 inhibit the cytochrome P450 (CYP) enzymes 3A4, 2C9, and 2C19. However, MBQ-168 is approximately 10× less potent than MBQ-167 at inhibiting CYP3A4, thus demonstrating its utility in relevant combination therapies. In conclusion, the MBQ-167 derivatives MBQ-168 and EHop-097 are additional promising antimetastatic cancer compounds with similar and distinct mechanisms.

**Significance:**

Targeting the related GTPases Rac and Cdc42 that regulate cancer metastasis is a viable strategy to impede metastasis of solid cancers. Herein, we describe new Rac and Cdc42 inhibitors with unique mechanisms and varying potency in different cancer cell lines. The MBQ-167 derivatives MBQ-168 and EHop-097 show promise as potential antimetastatic cancer agents.

## Introduction

Metastasis continues to be the leading cause of cancer-related deaths (1). Currently, there are no effective targeted therapeutics to prevent metastasis and reduce cancer mortality. Therefore, we are developing inhibitors to the homologous Rho GTPases Rac and Cdc42 that are pivotal regulators of the migratory and invasive steps of metastasis (2, 3). The central role of Rac and Cdc42 in multiple human cancers has been reviewed extensively (4–6). In breast cancer, Rac and Cdc42 dysregulation can be attributed to overexpression due to gene amplification and mRNA upregulation, hyperactivity via the upstream regulators guanine nucleotide exchange factors (GEF), overexpression of oncogenic cell surface receptors, or a combination of these (7, 8). Over 80 different GEFs have been elucidated, with the common mechanism of nucleotide exchange from the GDP-inactive bound state to a GTP-active bound state (9). GTP bound active Rac (1–3) and Cdc42 regulate multiple downstream signaling pathways, including P21-activated kinases (PAK), to promote cancer cell proliferation, survival, invasion, angiogenesis, and metastasis.

Therefore, much effort has been spent on developing Rac and Cdc42 inhibitors as anticancer compounds (8). Of the published Rac/Cdc42 targeting compounds, MLS000532223 and EHT1864 inhibit Rac activity by altering nucleotide binding to Rac; however, these inhibitors exhibit IC_50_s of >10 μmol/L (10, 11). Recently, Geneyus LLC reported an improved compound GYS32661, derived from EHT1864, which inhibits tumor progression and angiogenesis in mouse modals of renal and colorectal cancer. GYS32661 also inhibits Rac1B, the constitutively active form of Rac1, but with IC_50_s in the 1–3 μmol/L range (12). A majority of the available Rac inhibitors block the interaction of specific GEFs with Rac; albeit at IC_50_s in the high μmol/L range (13). The NSC23766 derivative AZA197 at 1–10 μmol/L inhibited the Cdc42-GEF Dbs activity and thus, colon cancer cell proliferation, and cancer progression in mice (14). Another Cdc42 selective inhibitor, ML141 has an IC_50_ ∼ 3–5 μmol/L, and inhibits melanoma cell migration (15). A recently characterized NSC derivative IODVA1 targets Rac and Cdc42, and significantly inhibits triple-negative breast cancer (TNBC) tumor growth in mice; however, this compound is effective only at μmol/L concentrations (16). In addition to these direct Rac/Cdc42 inhibitors, Ketorolac is an FDA-approved NSAID, where the S-enantiomer shows Rac1/Cdc42 inhibitor activity but at high μmol/L range. Even though R-ketorolac reduced ovarian cancer tumor burden in mice (17), it failed to significantly reduce tumor burden in a spontaneous breast cancer mouse model (MMTV-PyMT; ref. 18). The compounds MBQ-167, MBQ-168, and EHop-097, described herein, are promising anticancer agents due to their physiologically relevant IC_50_s and potent preclinical efficacy and safety in breast cancer cell and mouse models.

The most widely used Rac1 inhibitor in laboratory experiments is NSC23766, which inhibits Rac1 binding and activation by the GEFs Trio and Tiam1 (19). However, the high IC_50_ (>75 μmol/L) of NSC23766 limits its use as a therapeutic agent. Our group designed EHop-016, a derivative of NSC23766, which inhibits Rac activity with an IC_50_ of 1.1 μmol/L by blocking the interaction of Rac with the GEF Vav (20–22). Next, we characterized the improved EHop-016 derivative, MBQ-167, as a dual Rac1 and Cdc42 inhibitor with IC_50_ values of 103 and 78 nmol/L, respectively, in metastatic breast cancer cells. MBQ-167 inhibits PAK/LIM kinase/Cofilin signaling, lamellipodia extension, metastatic cancer cell polarity, migration, stem cell–like mammosphere formation, cell-cycle progression, and viability and induces apoptosis in metastatic cancer cells. Attempts to create MBQ-167 resistant cells were unsuccessful, due to the loss in cell polarity and loss of cell-substrate attachments to ultimately undergo apoptosis by anoikis mechanisms in 100% of breast cancer cells following 250 nmol/L MBQ-167 for 1 week. Consequently, MBQ-167 inhibits HER-2–positive (HER2^+^) tumor growth and metastasis to all organs by approximately 90% in immunocompromised mice (23). Moreover, in TNBC, MBQ-167 inhibits tumor growth, and spontaneous and experimental metastasis in immunocompromised and immunocompetent mouse models (24). In mice, MBQ-167 exhibits 35% bioavailability with half-lives of approximately 2.5 hours in plasma and approximately 8 hours in tumor tissue (25). These results highlight the specificity, potency, and bioavailability of MBQ-167, and support its clinical potential as a metastatic cancer therapeutic.

Even though MBQ-167 is our lead candidate for translational development, characterization of MBQ-167 derivatives is expected to identify a range of inhibitors, which may be effective in different cancer types that express various GEFs and are under the direction of multiple oncogenic upstream effectors. Therefore, we expect these new compounds to have cancer type or cell type specific activities. In the MBQ-167 derivatives, the carbazole group was maintained, which as a privileged scaffold, has been associated with anticancer, antibacterial, antifungal, and anti-inflammatory properties (26, 27). We hypothesized that by further exploring the structure-activity relationships based on MBQ-167, compounds with increased potency and improved biopharmaceutical properties could be obtained. Results show that several derivatives inhibit cancer cell viability and directed migration. Of these, MBQ-168 is further characterized as an anticancer compound with equal potency as MBQ-167 that can block GDP/GTP incorporation on Rac and Cdc42. We also report on the EHop-016 derivative Ehop-097 as a Vav/Rac specific inhibitor that is 10× more effective than the parent compound.

## Materials and Methods

### Synthesis of MBQ-167 Derivatives

The synthesis of the new derivatives was performed as described for MBQ-167 (23). Briefly, 3-azido-9-ethyl-9H-carbazole was reacted with the appropriate *in situ* prepared alkynyl Grignard reagent to form the 1,2,3-triazole ring. Therefore, the MBQ-167 9-ethyl-3-(1*H*-1,2,3-triazol-1-yl)-9*H*-carbazole core was maintained with substitutions or isosteric replacements on the 5-phenyl group (Table 1). In the second series, the 4-position is decorated with 4-(aryl)methanol substituents (Table 2). Quench of the obtained intermediate with aqueous ammonium chloride gave the compounds in Table 1. Quench of the intermediate with an aryl aldehyde gave the compounds in Table 2 as racemic mixtures. Details are provided in the Supplementary Materials and Methods.

**TABLE 1:** Structures of 1,5-disubstituted-1,2,3-triazole derivatives

Compound ID	1,5-disubstituted-1,2,3-triazoles	Name
**1a**	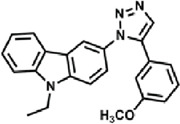	MBQ-168
**1b**	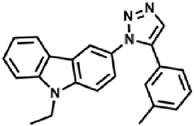	MBQ-169
**1c**	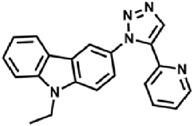	MBQ-170
**1d**	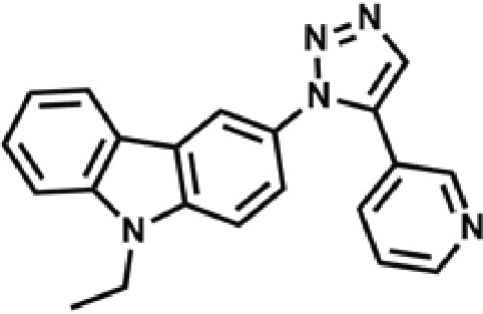	MBQ-171
**1e**	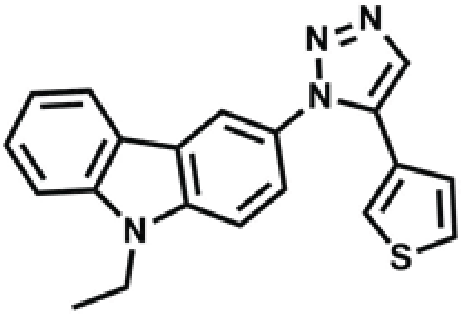	MBQ-172

**TABLE 2:** Structures of 1,4,5-trisubstituted-1,2,3-triazole derivatives

Compound ID	1,4,5-trisubstituted-1,2,3-triazoles	Name
**2a**	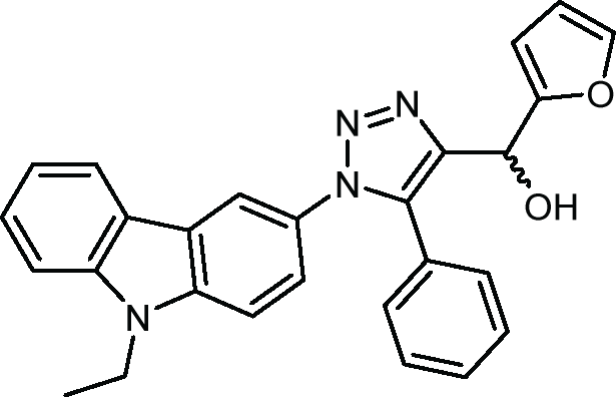	EHop-098
**2b**	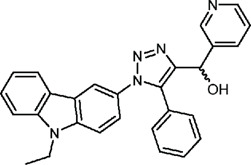	EHop-040
**2c**	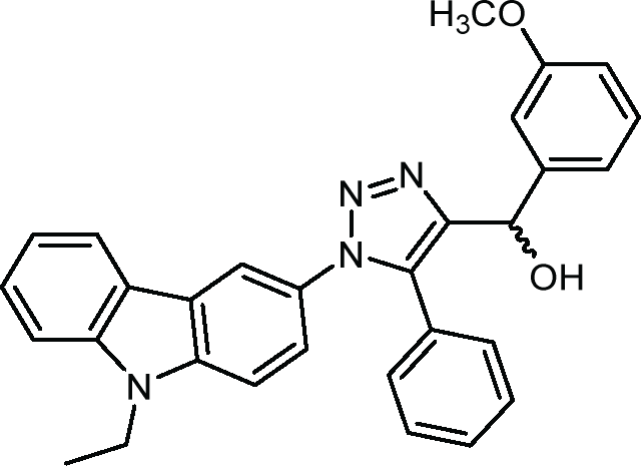	EHop-097

### Cell Culture

MDA-MB-231, MDA-MB-468, MDA-MD-157, MCF7, MCF10A, and human mammary epithelial cells (HMEC) were purchased from ATCC. GFP-tagged HER2-overexpressing metastatic cancer cell line (GFP-HER2-BM) characterized in ref. 28, was from Dr. Danny Welch, The University of Kansas Cancer Center, Kansas City, KS). All cell lines were cultured and maintained as described previously (20). All cell lines were authenticated, and *Mycoplasma* testing was routinely conducted. The GFP-HER2-BM, which is an independent early variant of the MDA-MB-435 cell line was authenticated by short tandem repeat profiling as a human breast cancer cell line and not a melanoma. For all experiments, cells from passage 2–15 were used, and cultured for a maximum of 3 months.

### Cell Viability Assay

Equal number of cells were seeded in a 24-well plate and incubated with vehicle or MBQ-167 derivatives at concentrations between 0 and 1,000 nmol/L for 72 and 120 hours. Using CellTiter 96 Non-Radioactive Cell Proliferation Assay (Promega, Corp.) according to manufactures’ instructions, formazan was used as an indicator of viable cells at an absorbance 570 nm in a Tecan Microplate Reader M100 (Tecan). Data were calculated as fold change from vehicle and transformed to logarithm base 10. 0 concentration was replaced with 1 nmol/L to fit using the 4-parameter logic nonlinear regression model in GraphPad Prism software. (*n* = 3 biologic replicates: with three technical replicates each).

### Apoptosis Assay

Equal number of cells were seeded in a 24-well plate and incubated with vehicle or MBQ-167 or derivatives at concentrations of 500 nmol/L for 48 hours. Apoptosis was measured using a Caspase-Glo3/7 Luminescence Assay Kit as per manufacturer's instructions (Promega, Corp.), as described previously (23). Briefly, Caspase-3/7 Glo reagent was added and incubated at room temperature for 60 minutes and caspase-3/7 activities were determined by detecting luminescence using Tecan Microplate Reader M100 (Tecan). Data were calculated as fold change from vehicle. (*n* = 3 biologic replicates: with three technical replicates each).

### Wound Healing Assay

MDA-MB-231 cells were plated on 12-well plates at equal cell density until confluent. Then the media was changed to 1% FBS, and a single scratch was made in the center of the monolayer culture with a pipet tip. Compounds were added immediately at 250 or 500 nmol/L and images were digitally acquired from a Keyence microscope (10×) at 0, 12, and 24 hours. The scratch area was quantified in ImageJ using the Macro Wound Healing Tool (http://dev.mri.cnrs.fr/projects/imagej-macros/wiki/Wound_Healing_Tool; *n* = 3 biologic replicates: with three technical replicates each).

### Phalloidin Staining and Fluorescence Microscopy

MDA-MB-231 cells growing on glass coverslips at low confluency were treated with vehicle, MBQ-167, MBQ-168 and EHop-097 at 250 nmol/L for 12 hours. Cells were fixed, permeabilized, and stained with rhodamine phalloidin to visualize F-actin as described previously (23). Fluorescence micrographs were acquired at 40× in a Keyence fluorescence microscope.

A549 lung cancer cells were serum starved and treated with 0–1,000 nmol/L MBQ-167, MBQ-168, or EHop-097 for 24 hours, and then treated with 200 ng/mL EGF for 5 minutes. Cells were fixed with 4% formaldehyde and F-actin stained with rhodamine phalloidin. Slides were visualized by fluorescence microscopy and five random fields were scored for the number of ruffles. Ruffle area was measured by thresholding for single intensity using ImageJ/Fiji software, as in ref. 29.

### Animal Protocol

All animal studies were conducted under approved protocol A8180118 Institutional Animal Care and Use Committee, in accordance with the NIH Guideline for the Care and Use of Laboratory Animals. Female SCID mice, 3–4 weeks old (Charles River Laboratories, Inc.), were maintained under pathogen-free conditions in High Efficiency Particulate Air (HEPA)-filtered cages.

#### Tumor Establishment

GFP-HER2-BM (∼7 × 10^5^) cells in Matrigel (BD Biosciences) were injected at the fourth right mammary fat pad under isoflurane inhalation (1%–3% in oxygen using an inhalation chamber at 2 L/minute) to produce orthotopic primary tumors. After tumor establishment (1-week after inoculation), animals were randomly divided into treatment groups (*n* = 5).

#### Administration of MBQ-167 and MBQ-168

Mice were treated with vehicle [12.5% ethanol (Sigma-Aldrich), 12.5% Cremophor (Sigma-Aldrich), and 75% 1X PBS, pH 7.4 (Corning)] or 5 mg/kg Body Weight (BW) MBQ-167 or MBQ-168 by intraperitoneal injection in a 100 mL volume 3× a week. Treatments continued until sacrifice at day 53.

#### Whole-body Fluorescence Image Analysis

Mammary tumor growth was quantified by integrated density of GFP fluorescence. After tumor establishment, mice were imaged before starting treatment, and once a week thereafter for 53 days, using the iBoxScientia (Analytik-Jena GmbH). Tumor fluorescence intensities were analyzed using ImageJ software (NIH, Bethesda, MD). Relative tumor growth was calculated as the integrated density of fluorescence of each tumor on each day of imaging relative to the integrated density of fluorescence of the same tumor on day 1 of treatment. After sacrifice at day 60, lungs, kidneys, livers, and spleens were excised and immediately stored in liquid N_2_. To quntify metastasis, stored organs were thawed and analyzed, as whole organs on both sides, using an Olympus MV10 fluorescence macro zoom system microscope and images acquired with an Olympus DP71 digital camera. The fluorescent lesions (green component of RGB images) were quantified for integrated density of fluorescent pixels using Image J software.

### Rac and Cdc42 Activation Assay

Breast cancer cells were seeded at equal density and treated with vehicle, MBQ-167 or MBQ-167 derivatives for 24 hours at various concentrations. Rac-GTP and Cdc42-GTP were detected using Rac Activity Assay or Cdc42 Activity Assay, as per manufacturer's instructions (Cell Signaling Technology). Briefly, total cell lysates were quantified and added equally to PAK-p21–binding domain conjugated Sepharose beads. After 1 hour of incubation, beads were washed, bound proteins extracted in sample buffer, and Rac1-GTP and Cdc42-GTP detected by Western blot analysis using Rac1, Rac1b, or Cdc42 antibody. (*n* = 3 biological replicates).

### Western Blots

Total cell lysates or pulldowns were Western blotted using routine procedures. The primary antibodies used were anti-Rac (Cell Signaling Technology), anti-Cdc42 (Abcam), anti-PAK1+PAK2+PAK3 (Cell Signaling Technology), anti-pPAK1 (T423)/PAK2 (T402; Abcam), anti-PAK1 + PAK2 + PAK3 (phospho T423; Abcam), Rac1B, and β-actin (Millipore-Sigma). Actin was used as a control; the Rac/Cdc42 inhibitors do not affect total actin expression, only its polymerization and organization of the cytoskeleton (23).

### Nucleotide Association Assay

The nucleotide loading on purified Rho GTPases was monitored using a fluorophore-based Rho GEF Exchange Assay Kit as per manufacturer's instructions (Cytoskeleton, Inc.). Drugs were diluted in exchange buffer and incubated with Rac1, Cdc42, or RhoA. The uptake of fluorescent nucleotide analog N-methylanthraniloyl-GTP (N-MAR-GTP) was measured as an increase in fluorescence due to binding to the nucleotide binding pocket of a GTPase. The fluorimeter was set at 20°C on kinetic mode and N-MAR GTP was detected at Ex 485 nm and Em 535 nm. After the initial five readings (T0), GEFs (Dbs or Vav2) or Ethylenediaminetetraacetic acid (EDTA) was added to stimulate exchange and readings were obtained every 30 seconds for at least 60 cycles.

### Rac1/Cdc42 (G15A) Pulldown Assay

MDA-MB-231 cells were lysed in 1% Triton X-100, 20 mmol/L HEPES, pH 7.4, 150 mmol/L NaCl, 5 mmol/L MgCl_2_, and protease inhibitors, and processed as described previously (20). Equal amounts of protein from cleared lysates were incubated for 1 hour at 4°C with glutathione-agarose beads conjugated to GST-Rac1 and GST-Cdc42(G15A) nucleotide-free mutant (Cell Biolabs) that were preincubated (for 1 hour) with vehicle or Rac/Cdc42 inhibitors, as indicated. The beads were washed, and pulldowns were immunoblotted for Vav2 or Ect2.

### CyP450 Enzyme Assay

The assay was carried out using P450-Glo Screening System (Promega). The system contained recombinant human CYP enzyme and P450 reductase (and cytochrome b5 for CYP2C9, 2C19, and 3A4), luminogenic substrates appropriate for each CYP enzyme, a NADPH regeneration system (containing NADP+), and luciferin detection reagent. Control membranes devoid of CYP activity were used as negative controls. Ketoconazole, troglitazone and sulfaphenazole were used as positive controls. These compounds and organic solvents acetonitrile, and DMSO were purchased from Sigma-Aldrich.

First, 12.5 μL of the 4X stock solution of test compounds or positive controls (appropriate for each enzyme) were added to the “treated” wells. For the “untreated” (the values from these wells represent total CYP activity) and “minus-P450 control” wells (the values from these wells represent the CYP-independent background luminescence of the assay), 12.5 μL of vehicle was added. Then, 12.5 μL of the 4X control reaction mixture (containing membrane preparations devoid of CYP enzymes, the appropriate luminogenic substrate, and potassium phosphate buffer) were added to the minus-P450 control wells, and 12.5 μL of the 4X reaction mixture (containing human CYP membrane preparations, the appropriate luminogenic substrate, and potassium phosphate buffer) were added to all other wells. The plates were preincubated at room temperature for 10 minutes with the reaction mixture (membrane with enzyme, substrate, and 1 mol/L potassium phosphate buffer), and then 2X NADPH regeneration system was added to initiate the reaction. After incubation at room temperature for 30 minutes, 50 μL of the Luciferin Detection Reagent was added to stop the reaction and generate the luminescent signal. Before reading, the plate was incubated at room temperature for 20 minutes to stabilize the luminescent signal. The luminescence was measured using an Infinite F200 plate reader (Tecan). The values were displayed as relative light units. The percentage of CYP enzyme activity versus log concentration of test compounds were plotted to calculate the IC_50_ values.

### Statistical Analysis

All half maximal growth inhibitory concentration (GI_50_) values were determined by using the 4-parameter logistic curve model in GraphPad Prism 9 software. Statistical analyses were done also using GraphPad Prism 9 software, and differences were considered statistically significant at *P* ≤ 0.05 using ordinary one-way ANOVA or unpaired Student *t* test with Dunnet multiple comparison tests.

### Data Availability

The data generated in this study are available within the article and its Supplementary Data.

## Results

### MBQ-167 Derivatives Inhibit Cell Viability and Apoptosis in Metastatic Cancer Cells

**FIGURE 1:**
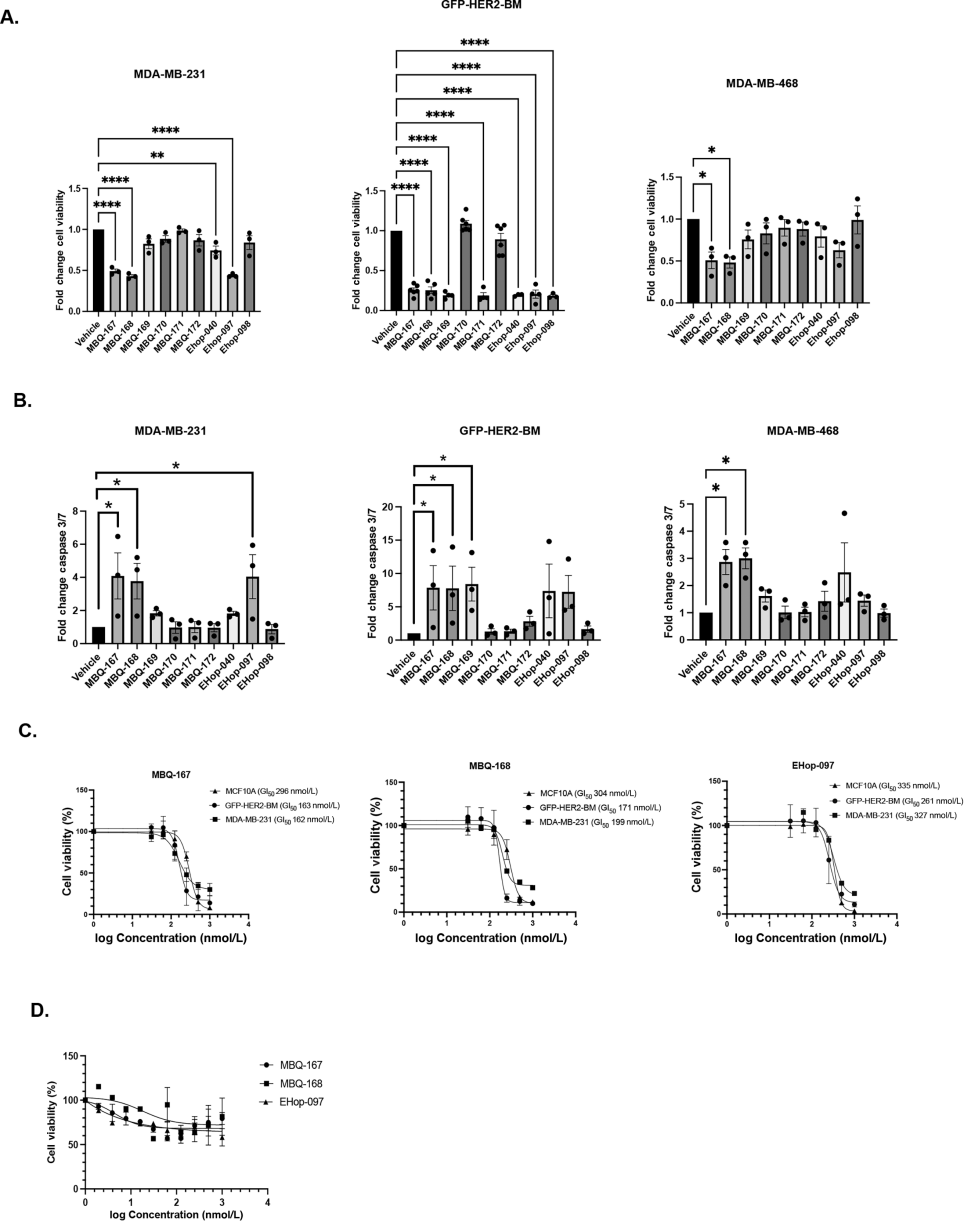
MBQ-167 derivatives decrease cancer cell viability and induce cell death through apoptosis. **A,** Effect of MBQ-167 derivatives on viability of MDA-MB-231, GFP-HER2-BM, and MDA-MB-468 cells. Cells plated at equal density were treated with 500 nmol/L of compounds. After 72 hours, cells viability was measured by a MTT (3-[4,5-dimethylthiazol-2-yl]-2,5 diphenyl tetrazolium bromide) assay. The bar graphs are presented as individual fold change datapoint from vehicle and grouped mean ± SEM. (*n* = 3–5; *, *P* < 0.05; **, *P* < 0.01; ****, *P* < 0.0001). **B,** Effect of MBQ-167 on caspase-3/7 activities for MDA-MB-231, GFP-HER2-BM, and MDA-MB-468 cells. Cells plated at equal density were treated with 500 nmol/L of compounds. After 48 hours, caspase-3/7 activity was measured by luminescence. The bar graphs are presented as individual fold change datapoints from vehicle and groups, mean ± SEM. (*n* = 3; *, *P* < 0.05). **C,** Percentage cell viability of MDA-MB-231, GFP-HER2-BM, or MCF-10A cells with MBQ-167, MBQ-168 or EHop-097 at concentrations ranging from 0 to 1,000 nmol/L. **D,** Percentage cell viability of HMEC cells following MBQ-167, MBQ-168 and EHop-097. Cells were treated for 120 hours at concentrations ranging from 0 to 1,000 nmol/L. GI_50_ curves for percentage cell viability are relative to vehicle from three biologic replicates each with two technical replicates. Four-parameter dose–-response curves generated using GraphPad Prism are shown.

The new derivatives of MBQ-167 listed in Tables 1 and 2 were successfully synthesized to 98% purity. The effect of these derivatives, on cancer cell viability at 500 nmol/L, was tested in GFP-HER2-BM, MDA-MB-231, and MDA-MB-468 cancer cell lines. MBQ-167 and MBQ-168 significantly inhibited the viability of all three cell lines by approximately 25%–50% at 72 hours. Although EHop-097 significantly inhibited MDA-MB-231 cell viability by 50%, this was a nonstatistically significant trend in the more aggressive EGFR (EGFR++) TNBC cell line MDA-MB-468. The compounds MBQ-167, MBQ-168, MBQ-169, and MBQ-171 were more potent in the metastatic GFP-HER2-BM cells as evidenced by an approximately 75% inhibition. In this cell line, EHop-040, -097, and -098 inhibited cell viability by 80% (Fig. 1A). Therefore, MBQ-168 and EHop-097 appear to have equal efficacy as MBQ-167 in reducing TNBC cell viability, while additional MBQ-167 derivatives were active in the GFP-HER2-BM cell line that we have previously shown to have high Rac activity (30).

Because the Rac/Cdc42 inhibitor MBQ-167 induces cell-cycle arrest and subsequent apoptosis, we tested the effect of the MBQ-167 derivatives on apoptosis via analysis of caspase-3/7 activity in cancer cell lines. The MBQ-167 derivatives that inhibited cell viability, that is, MBQ-168 and EHop-097 in the MDA-MB-231 cell line, MBQ-168, MBQ-169, and EHop-040, and EHop-097 in the GFP-HER2-BM cell line, and MBQ-168 and EHop-040 in the MDA-MB-468 cell line, significantly increased Caspase-3/7 activity. MBQ-168 and Ehop-097 induced the activity of caspase-3/7 three to five times more compared with the vehicle, similar to MBQ-167 (Fig. 1B). Therefore, MBQ-168 and Ehop-097 were selected for further analysis in the MDA-MB-231 TNBC cell line.

The GI_50_ of compounds MBQ-168 and EHop-097 were analyzed and compared with MBQ-167. As expected, MBQ-167 treatment for 120 hours resulted in GI_50_s of approximately 160 nmol/L for GFP-HER2-BM and MDA-MB-231 cells, while the GI_50_ of the noncancer transformed cell line MCF10A was higher at 296 nmol/L (Fig. 1C). MBQ-168 demonstrated a similar effect with GI_50_s of 171 nmol/L in GFP-HER2-BM, 199 nmol/L in MDA-MB-231 cells, and 304 nmol/L in MCF10A cells. The structurally distinct EHop-097 demonstrated higher GI_50_s in GFP-HER2-BM (261 nmol/L) and MDA-MB-231 (327 nmol/L) cells, which were in the same range as the GI_50_ of 335 nmol/L for the MCF10A cell line. MBQ-167, MBQ-168, or EHop-097 did not affect the viability of HMECs indicating that these compounds are nontoxic to normal epithelial cells (Fig. 1D).

As shown in Supplementary Fig. S1, when the viability of all cell lines was compared with vehicle at a fixed concentration of 250 nmol/L, MBQ-167 and MBQ-168 were not as potent in the ER/PR-positive MCF7 breast cancer cells and the noncancer MCF10A cells, demonstrating a 40% and 30% inhibition of cell viability, compared with 50% inhibition in MDA-MB-231 cells and approximately 85% in GFP-HER2-BM cells. EHop-097 was not effective in the MDA-MB-231, MCF7, or MCF-10 cell lines at 250 nmol/L, but inhibited GFP-HER2-BM cells by approximately 60% at this concentration. Therefore, MBQ-167 derivatives demonstrated cell type–dependent effects on cancer cell viability with a more cytotoxic effect on metastatic cancer cell lines.

### MBQ-168 and Ehop-097 Inhibit Breast Cancer Cell Migration and Cell Polarity

**FIGURE 2:**
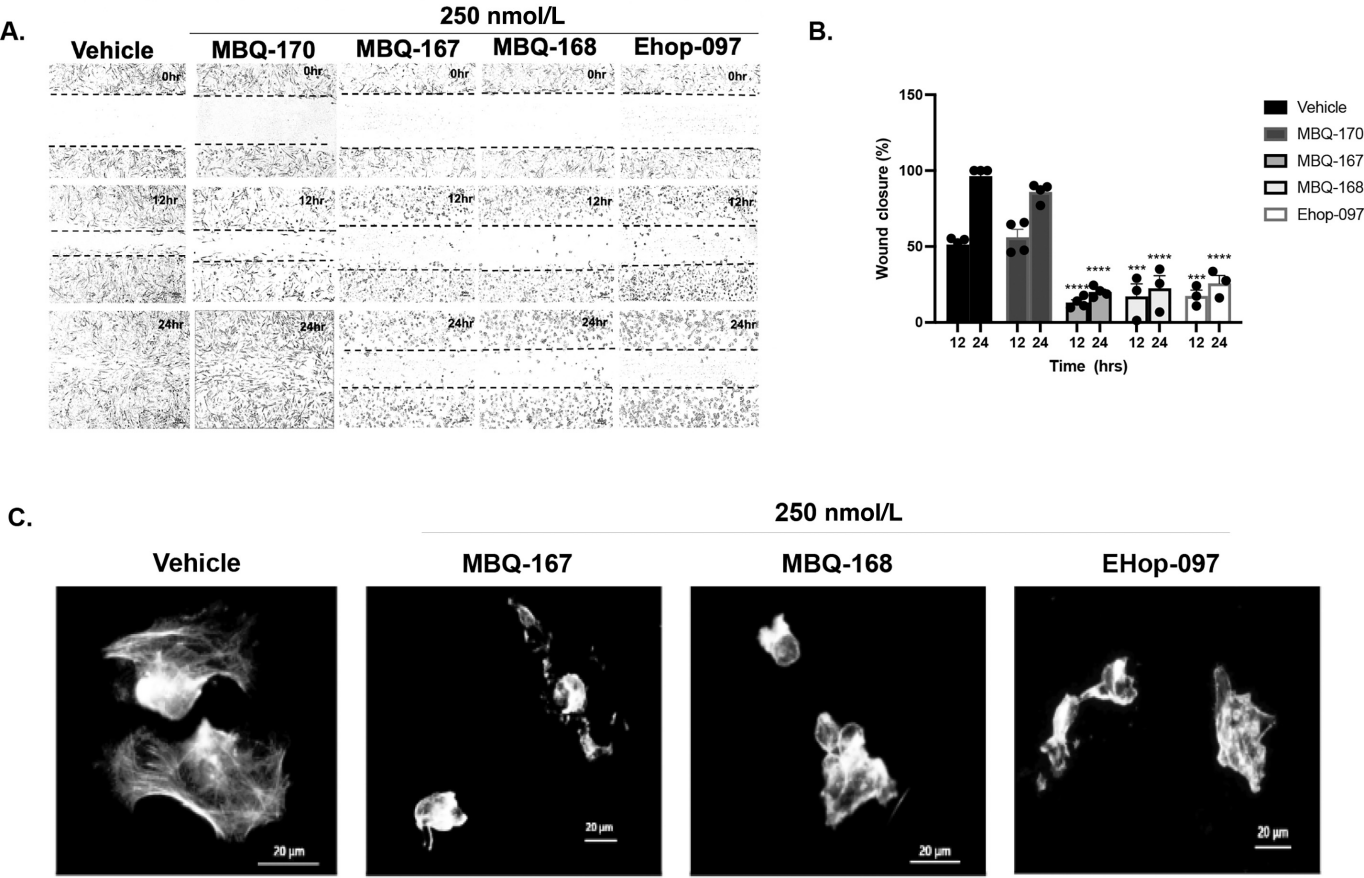
MBQ-167 derivatives inhibit cell migration in TNBC cells. **A,** Effect of MBQ-167 on cell migration in a wound healing assay. MDA-MB-231 cells plated at equal density were subjected to a scratch in the center and treated with MBQ-170, MBQ-167, MBQ-168, and Ehop-097 at 0, 250, or 500 nmol/L. Micrographs were digitally acquired at 0, 12, and 24 hours and the area of the wound quantified for each treatment and presented relative to the distance at time 0. **B,** Percentage of wound closure for vehicle, MBQ-170, MBQ-167, MBQ-168, and Ehop-097 treatments. The bar graphs are presented as individual fold change datapoints from vehicle and grouped mean ± SEM. (*n* = 3–4; ***, *P* < 0.001; ****, *P* < 0.0001). **C,** Breast cancer cell phenotype following MBQ-167, MBQ-168, or Ehop-097 treatment. MDA-MB-231 cells were treated with 0 or 250 nmol/L MBQ-167, MBQ-168, or Ehop-097 for 12 hours and fixed and stained with rhodamine phalloidin. Representative fluorescent micrographs are shown.

Wound healing assays were used to test the antimigratory effect of MBQ-167 derivatives in MDA-MB-231 TNBC cells. After generation of the wound in a confluent cell layer of serum-starved cells, 250 nmol/L of MBQ-167, MBQ-168 or Ehop-097 were added in serum plus media, and the area of the wound quantified from digital images at 0, 12, and 24 hours. Results show that the wound was closed in 24 hours in vehicle or MBQ-170–treated cells, where MBQ-170 was selected as a negative control, since this compound did not affect the viability of cancer cells. In contrast, the wound area was maintained at 12 and 24 hours for cells treated with MBQ-167, MBQ-168, or EHop-097, in a statistically significant manner compared with vehicle but not to MBQ-167 (Fig. 2A and B). MBQ-167 induces detachment of metastatic cancer cells from the substrate to ultimately undergo anoikis, as was reported for MDA-MB-231, GFP-HER2-BM, and MDA-MB-468 cells (24). Thus, MDA-MB-231 cells were treated with MBQ-167, MBQ-168, or Ehop-097 for 12 hours at 250 nmol/L, and the actin cytoskeleton was stained with rhodamine phalloidin to identify changes in F-actin, which are required for cell migration. Figure 2C shows the cell phenotype, where vehicle cells are spread with an actin cytoskeleton organized into stress fibers and membrane ruffles. However, MBQ-167–treated or MBQ-168–treated cells acquired a round shape with punctate actin staining following 12 hours of treatment. Nevertheless, these unattached cells are still viable until >24 hours treatment, as ascertained by viability assays in Fig. 1 at 72 hours. EHop-097–treated cells were smaller and less spread but did not have the characteristic loss of polarity induced by the MBQ compounds. We also observed similar cell rounding in metastatic GFP-HER2-BM cells. When attached and detached GFP-HER2-BM cells were separated and analyzed for directed migration using a Transwell assay, both cell types were inhibited by MBQ-168, similar to what was reported for MBQ-167 (Supplementary Fig. S2).

To determine whether the effect on the actin cytoskeleton was limited to TNBC cells and the HER2^+^ metastatic cancer cell line, the effects of MBQ-167 and MBQ-168 were tested in the lung adenocarcinoma cell line A549, following stimulation with EGF to induce actin-based ruffles that are under Rac/PAK regulation. Treatment with MBQ-167, MBQ-168, or EHop-097 at 0–1,000 nmol/L resulted in a loss of actin cytoskeletal extensions where both ruffle number and area in response to EGF were significantly reduced compared with vehicle treatments, in a concentration-dependent manner. At 500 nmol/L, MBQ-167 induced a 54%, MBQ-168 a 67%, and EHop-097 a 33% loss in ruffles. At a higher concentration of 1,000 nmol/L, the percentage decrease in response to MBQ-167 was 63%; MBQ-168, 79%; and EHop-097, 58% (Supplementary Fig. S3A and S3B). Therefore, MBQ-168 appears to be more effective at inhibiting actin-based motile structures compared with MBQ-167 and EHop-097 in metastatic lung cancer cells, even though we did not see significant differences among MBQ-167 and MBQ-168 on the actin cytoskeleton of metastatic breast cancer cells.

### MBQ-168 Inhibits Mammary Tumor Growth and Metastasis in a Mouse Model

**FIGURE 3:**
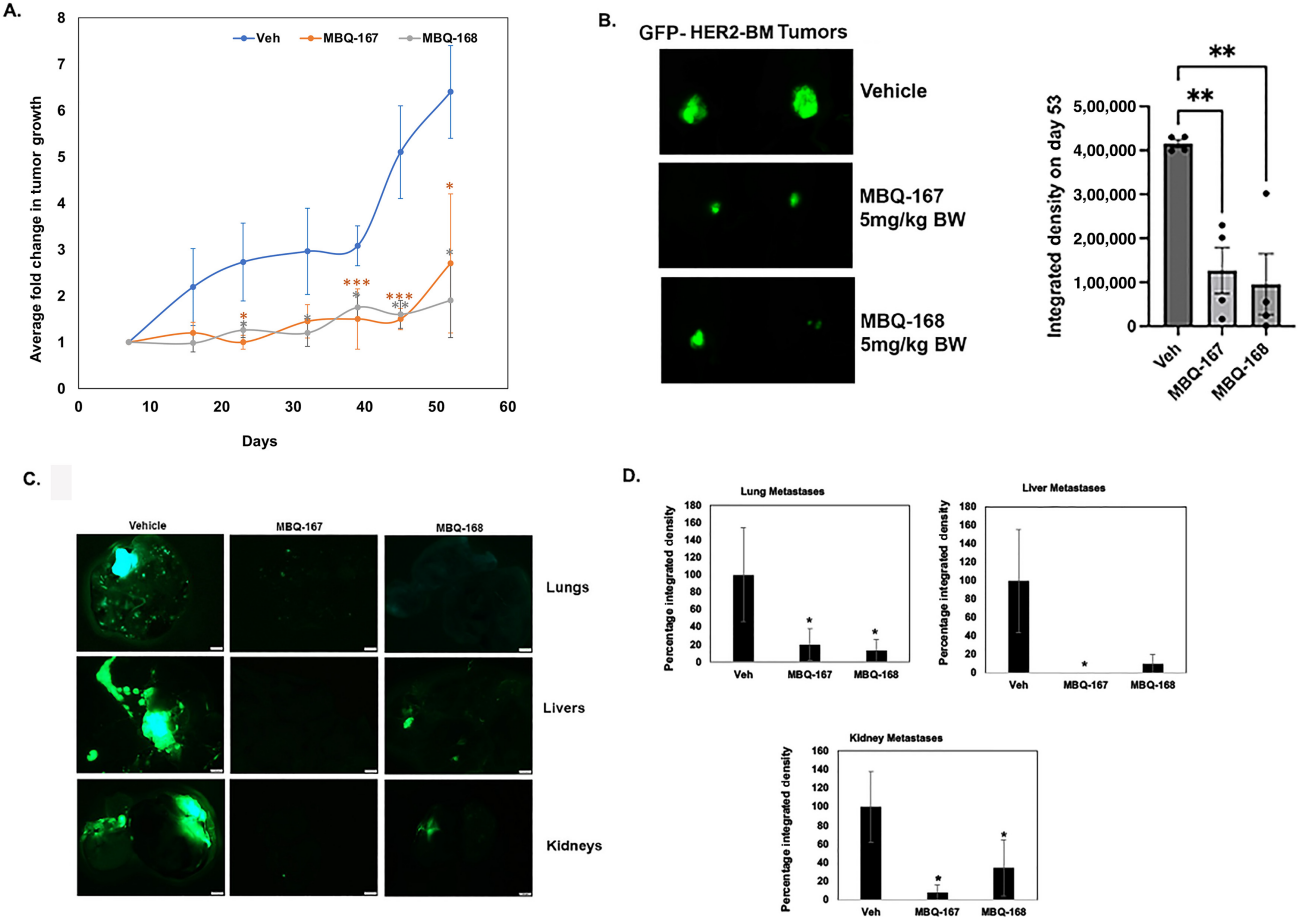
MBQ-167 and MBQ-168 demonstrate similar antitumor and antimetastasis effects in a HER2^+^ metastatic cancer mouse model. GFP-HER2-BM metastatic cancer cells were used to establish mammary fatpad tumors in SCID mice. When the tumors reached approximately 100 mm^3^, vehicle (12.5% ethanol, 12.5% Cremophor and 75% 1× PBS, pH 7.4), or 5 mg/kg BW MBQ-167 or MBQ-168 were administered by intraperitoneally (100 μL each) 5× a week for 53 days. **A,** Average tumor growth was quantified by fluorescence image analysis using Image J, and relative tumor growth was calculated as the integrated density for each tumor on each day of imaging (1× a week 2 months) divided by its integrated density on day 1. **B,** Left, Fluorescence images of representative tumors (2/treatment) at day 53. Right, Integrated density of vehicle-treated, MBQ-167–treated, or MBQ-168–treated tumors on day 53 (*n* = 5 ± SEM). At necropsy, lungs, livers, and kidneys were removed and whole organs imaged for fluorescent metastatic foci. **C,** Representative organs under fluorescence microscopy for 5 mg/kg BW of MBQ-167 and MBQ-168 treatment. **D,** Average integrated density of fluorescent metastatic foci/organ. *n* = 5 ± SEM. *, *P* < 0.05.

To determine whether the antiviability, antisurvival, and antimigration effects of MBQ-168 resulted in anticancer effects *in vivo*, we determined the effect of MBQ-168 on breast cancer progression in an orthotopic spontaneous metastasis mouse model. The metastatic GFP-HER2-BM cells were used to establish mammary fatpad tumors in SCID mice and treated with vehicle, or 5 mg/kg BW MBQ-167 or MBQ-168. Mammary tumor progression was analyzed by fluorescence image analysis. Results show similar effects for both MBQ-167 and MBQ-168 with approximately 90% inhibition in tumor growth compared with vehicle (Fig. 3A and B). Next, we analyzed the effect of MBQ compounds on metastasis by quantifying fluorescent metastatic foci from excised lungs, livers, and kidneys. Again, approximately 90% inhibition of metastases was observed for lung, liver, and kidney metastases from mice treated with both MBQ-167 and MBQ-168 compared with vehicle (Fig. 3C and D). Therefore, MBQ-168 acts similar to MBQ-167 to inhibit both tumor growth and metastasis of GFP-HER2-BM mammary tumors, as reported before for MBQ-167 (24). We did not observe appreciable brain metastases from this spontaneous metastasis mouse model to be statistically relevant, even though HER2^+^ breast cancers are known to metastasize to the brain in human patients. Potential toxicity was analyzed by phenotypic appearance of the mice, weight, and liver enzymes from plasma, as indicators of liver toxicity. Good Laboratory Practices (GLP) data from a Contact Research Organization demonstrated no apparent toxicity from MBQ-167 in rodents or dogs up to 1,000 mg/kg. Supplementary Figure S4 demonstrates that similar to MBQ-167, MBQ-168 treatment 5× a week at 5 mg/kg for 53 days did not significantly change weight or liver toxicity, compared with vehicle controls, as measured from aspartate transaminase and alanine transaminase levels from plasma.

### MBQ-168 Inhibits Rac1/Rac1b/Cdc42/PAK Activation in Breast Cancer Cells

Because MBQ-168 is structurally and functionally similar to MBQ-167, we analyzed its mechanism of action as a Rac/Cdc42 inhibitor. Using pulldown assays via the p21-binding domain of PAK to isolate the GTP bound active Rac or Cdc42, we compared the efficacy of MBQ-167 to inhibit Rac and Cdc42 activation. First, the metastatic GFP-HER2-BM cell line was used to analyze the effect of 250 nmol/L MBQ-167 or MBQ-168 when attached and detached cells were collected following 24 hours incubation and subjected to pulldown assays for active Rac.GTP. Similar to MBQ-167, MBQ-168 affects cell polarity to result in cell detachment from the substrate to ultimately undergo apoptosis, that is, anoikis. We observed a complete depletion of Rac1-GTP bound MBQ-168 in both attached and detached cell population at 250 nmol/L in the metastatic GFP-HER2-BM cells. Notably, unlike with MBQ-167, Rac1 expression was approximately 100% decreased in the detached cell population in response to MBQ-168 treatment. As seen in the control Western for actin, which is also depleted, MBQ-168 appears to be a more potent apoptosis inducer and the detached cell population goes through anoikis at a faster rate than following MBQ-167 treatment. Furthermore, MBQ-168 was also more effective than MBQ-167 in reducing Rac1 activation (Rac1.GTP) in the attached cell population by approximately 100% in the GFP-HER2-BM cell line (Supplementary Fig. S5).

**FIGURE 4:**
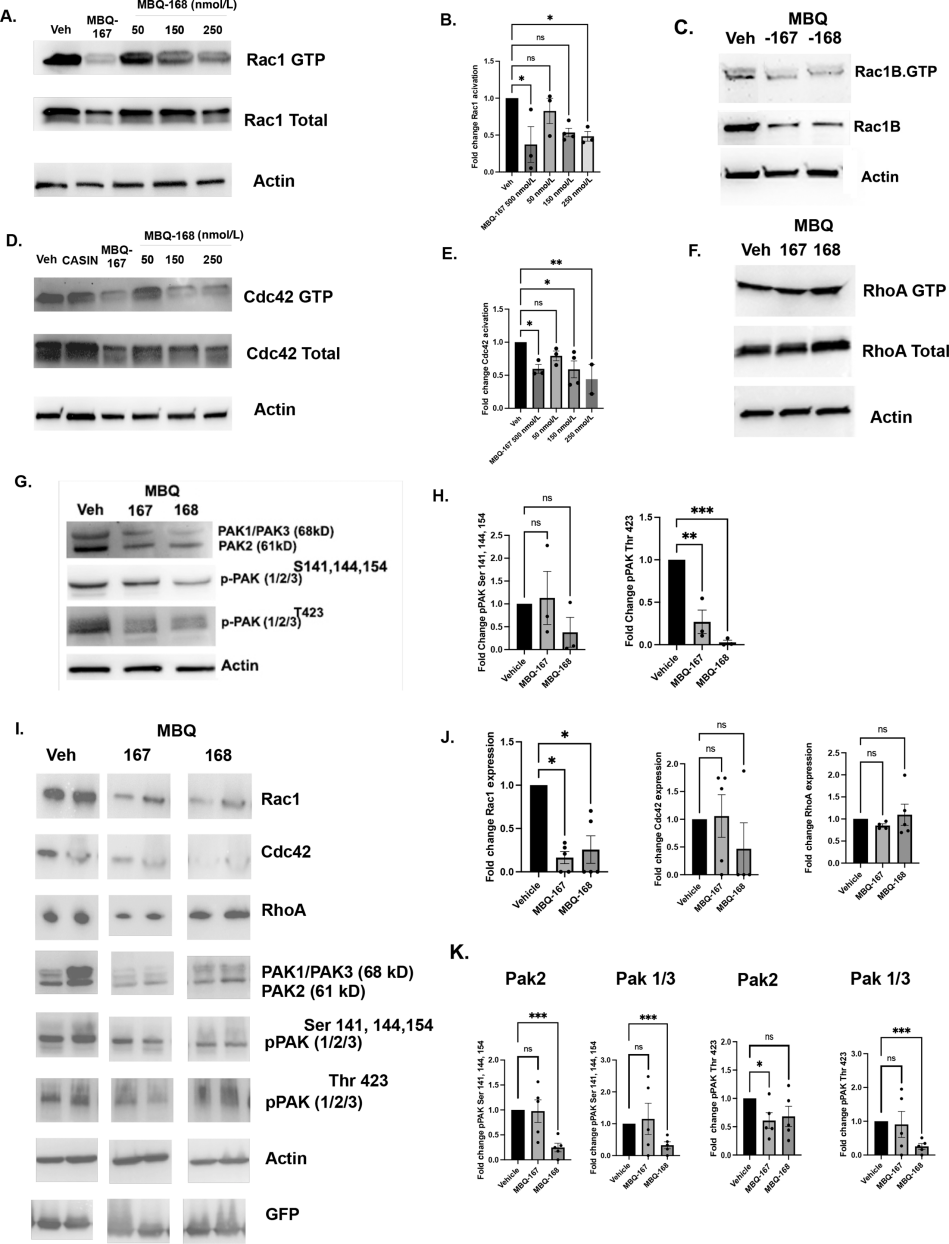
Effect of MBQ-167 and MBQ-168 on Rac/Cdc42/PAK activation. MDA-MB-231 cells were treated for 24 hours with various concentrations of MBQ-167 and MBQ-168. Cells were lysed and equal amounts of proteins were subjected to pulldown assays using the p21-binding domain of PAK to isolate the GTP bound Rac1 or Cdc42. **A,** Representative Western blot analysis for cell lysates following 50, 150, and 250 nmol/L MBQ-168 or 500 nmol/L of MBQ-167 Western blotted with antibodies to Rac1. Top, Pulldown showing GTP bound Rac1; Middle, Total Rac1 in lysates; Bottom, Actin as a loading control. **B,** Right, Results from positive bands in Western blots quantified using ImageJ. Rac1 activity for each MBQ-167 and MBQ-168 treatment was divided by the vehicle controls for each experiment to obtain relative Rac1(*n* = 3–4; *, *P* < 0.05; ns, not significant). **C,** Representative Western blot analysis for Rac1B following 500 nmol/L MBQ-167 or MBQ-168 for 24 hours, in the MDA-MB-468 cell line, which expresses the Rac1B splice variant. Top band, Rac1B.GTP from the pulldown; Middle band, Total Rac1B from cell lysates; Bottom band, Actin (*N* = 3). **D,** Representative Western blot analysis for cell lysates following 50, 150, and 250 nmol/L MBQ-168, or 500 nmol/L of MBQ-167 Western blotted with antibodies to Cdc42. Top, Pulldown showing GTP bound Cdc42; Middle, Total Cdc42 in lysates; Bottom, Actin as a loading control. **E,** Right, Results from positive bands in Western blots quantified using ImageJ. Cdc42 activity for each MBQ-167 and MBQ-168 treatment was divided by the vehicle controls for each experiment to obtain relative Cdc42. (*n* = 3–4; *, *P* < 0.05; **, *P* < 0.01; ns, not significant). Error bars represent SEM. **F,** Effect of MBQ-167 and MBQ-168 on Rho activation. Rho activation assay was done using the Rho.GTP-binding domain of Rhotekin. **G,** Effect of MBQ-167 and -168 on PAK activation. MDA-MB-231 cells were treated with vehicle, or 250 nmol/L MBQ-167 or MBQ-168 for 12 hours. Cells were lysed and Western blotted for total and active PAK. Left, Top, Cell lysates probed with an anti PAK1/2/3 antibody; second, Western blot analysis with anti phospho (p)-PAK (1/2/3)^Ser141,144,154^; third, Western blot analysis with p-PAK (1/2/3)^Thr423^; fourth, Anti actin. **H,** Fold change in p-PAK/PAK for PAK 1/3 (68 kD) for total p-PAK^Ser141,144,154^ and p-PAK^Thr423^. (*n* = 3; **, *P* < 0.01; ***, *P* < 0.001; ns, not significant). **I,** Tumors from the study in Fig. 3 where SCID mice were treated 5× a week with vehicle or 5 mg/kg MBQ-167 or MBQ-168 for 53 days were isolated at necropsy and the proteins extracted by lysis. Tumor lysates were Western blotted with antibodies specific for Rac1, Cdc42, PAK1/2/3, p-PAK (1/2/3)^Ser141,144,154^, or p-PAK^Thr423^. Lysates from two tumors for each treatment are shown (*n* = 5). **J,** Fold change in Rac1, Cdc42, and RhoA expression from tumor lysates (*n* = 5; *, *P* < 0.05; ns, not significant). **K,** Fold change in P-PAK/PAK for PAK 1/3 (68 kD) and PAK2 (61 kD) bands for PAK1/2/3 antibodies specific for total and P-^Ser141,144,154^ and p-PAK^Thr423^. (*n* = 5; *, *P* < 0.05; ***, *P* < 0.001; ns, not significant). The bar graphs are presented as individual fold change datapoint from vehicle and grouped mean ± SEM.

Therefore, we only analyzed Rac1 activation in the attached cell population in MDA-MB-231 cells following 24 hours in 250 nmol/L MBQ-167 or MBQ-168, where incubation times longer than 24 hours are required for cell detachment and subsequent anoikis. Figure 4A and B demonstrates that MBQ-167 and MBQ-168 inhibits Rac.GTP incorporation in a dose-dependent manner. Moreover, at concentrations >500 nmol/L, MBQ-167 also reduces total Rac expression. In addition to the close isoforms Rac1,2,3, Rac1B is a splice variant with an additional exon (exon 3b) of 19 amino acids. Biochemically, this in-frame insertion of exon 3b results in an accelerated GDP/GTP exchange and an impaired GTP hydrolysis compared with Rac1 (31). Therefore, we tested whether MBQ-167 and derivatives also inhibited Rac1B activation. In the MDA-MB-468 cell line, which expresses the oncogenic Rac1B splice variant (32), 500 nmol/L of MBQ-167 or MBQ-168 inhibited activation of Rac1B, as well as expression, without affecting actin expression. MBQ-167 was more effective than MBQ-168 at inhibiting Rac1B activity, demonstrating a 30% inhibition of Rac1B at 250 nmol/L (Supplementary Fig. S6A) with an IC_50_ of 500 nmol/L, which is 5× higher than the IC_50_ for Rac1 inhibition (Fig. 4C). Notably, MBQ-167 also inhibits the (P29S) oncogenic Rac1 mutation, as shown in Supplementary Fig. S6B.

When the effect of MBQ-168 was compared with MBQ-167 in Cdc42 activation, we found similar results as MBQ-167, where Cdc42 activity was reduced in a dose-dependent manner in response to MBQ-168 for 24 hours (Fig. 4D and E). Similarly, both compounds do not affect the related GTPase Rho activation at 250 nmol/L (Fig. 4F).

To determine whether downstream signaling from Rac and Cdc42 were also inhibited by MBQ-168, we analyzed the phosphorylated state of its downstream effector, PAK1/2/3. MDA-MB-231 cells were treated with 250 nmol/L MBQ-167 or MBQ-168 for 24 hours, lysed and Western blotted for PAK using an antibody that detects group I PAKs (PAK1,2,3). The lysates were also probed for phosphorylation state of PAK 1/2/3 at the residues of threonine (Thr) 423 and serine (Ser) at S141 (PAK1), S144 (PAK2), and S154 (PAK3; Fig. 4G). For PAK1/3, detected as a 68 kD band, MBQ-167 decreased p-PAK ser 141, 154 by approximately 60% and MBQ-168 by approximately 95%. Similarly, p-PAK1/3 levels were reduced approximately 75% by MBQ-167 and approximately 100% by MBQ-168. For PAK2, which was detected as a 61 kD band in Western blot analysis, both compounds only reduced p-PAK2 S144 by approximately 50%, while p-PAK2^T423^ was reduced by approximately 60% in response to MBQ-167 and approximately 100% by MBQ-168 (Fig. 4H). Therefore, MBQ-168 appears to be a more effective PAK inhibitor than MBQ-167.

This inhibition in PAK expression followed by a more potent inhibition of phosphorylation in activation residues were also observed from tumor extracts. Tumors from the experiment shown in Fig. 3 where mice bearing GFP-HER2-BM tumors were treated with 5 mg/kg BW MBQ-167 or MBQ-168 for 53 days, were lysed and subjected to Western blotting for Rac, Cdc42, RhoA, PAK, and p-PAK (Fig. 4I). Decreases were observed in total Rac, and PAK1/2/3 levels, while Cdc42 and RhoA remain unaffected in the tumors following prolonged treatment with MBQ-167 or MBQ-168 (Fig. 4J) The tumors from mice that received MBQ-167 treatment did not show changes in p-Serine residues 141,144,154; however, MBQ-168 significantly reduced p-Serine levels in PAK1, 2, and 3 by approximately 75%. In PAKs 1, 2, 3, Thr 423 phosphorylation was reduced compared with vehicle treatments for both MBQ-167 and MBQ-168 treatments by about approximately 50% (Fig. 4K). These data indicate that the reduction in tumor growth and metastasis demonstrated by the mice treated with MBQ-167 or MBQ-168 may be attributed to reduced Rac/Cdc42/PAK activities.

### EHop-097 is a Vav/Rac Inhibitor

**FIGURE 5:**
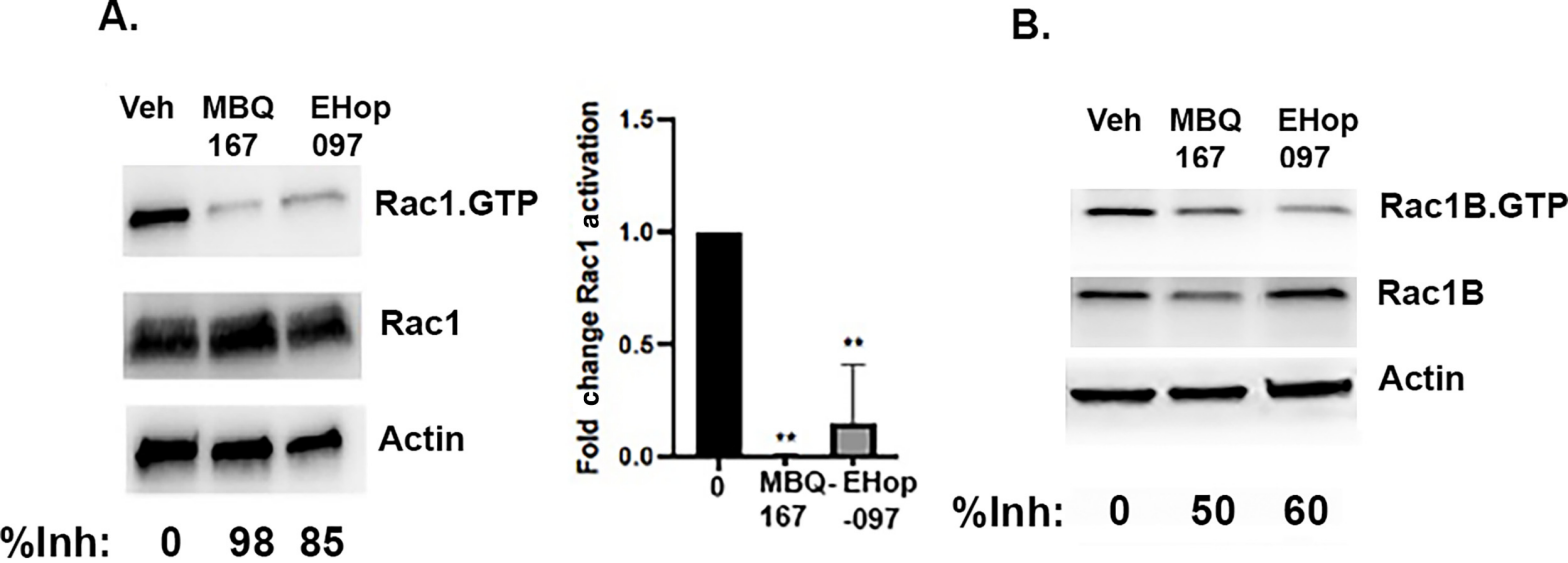
Effect of EHop-097 on Rac1 and Rac1B activation. **A**, MDA-MB-231 TNBC cells were treated for 24 hours with vehicle or 250 nmol/L MBQ-167. Cells were lysed and equal amounts of proteins were subjected to pulldown assays using the p21-binding domain of PAK to isolate the GTP bound Rac1 or Rac1B and Western blotted with specific antibodies for Rac1 (21 kD band). Left, representative Western blot analysis, top, pulldown showing GTP bound Rac1; second, Total Rac1 in lysates; third, actin as a loading control. Right, Quantification of positive bands in A. *N* = 3. **B,** MDA-MB-468 TNBC cells were treated for 24 hours with vehicle, 500 nmol/L MBQ-167 or EHop-097. Lysates of equal protein concentrations were used to pulldown active Rac1B.GTP. Representative Western blot probed with antiRac1B (23 kD band) or actin is shown. Top, Rac1B.GTP from pulldown; second, Rac1B from input; and third, Actin (*N* = 3).

Like MBQ-167, 250 nmol/L EHop-097 significantly inhibited Rac1 activity by approximately 85% in MDA-MB-231 TNBC cells (Fig. 5A). When tested for the inhibition of activation of the Rac1B splice variant in MDA-MB-468 TNBC cells, EHop-097 also acted similar to MBQ-167 by an approximately 60% inhibition, demonstrating its utility in inhibition of an oncogenic form of Rac1 (Fig. 5B).

**FIGURE 6:**
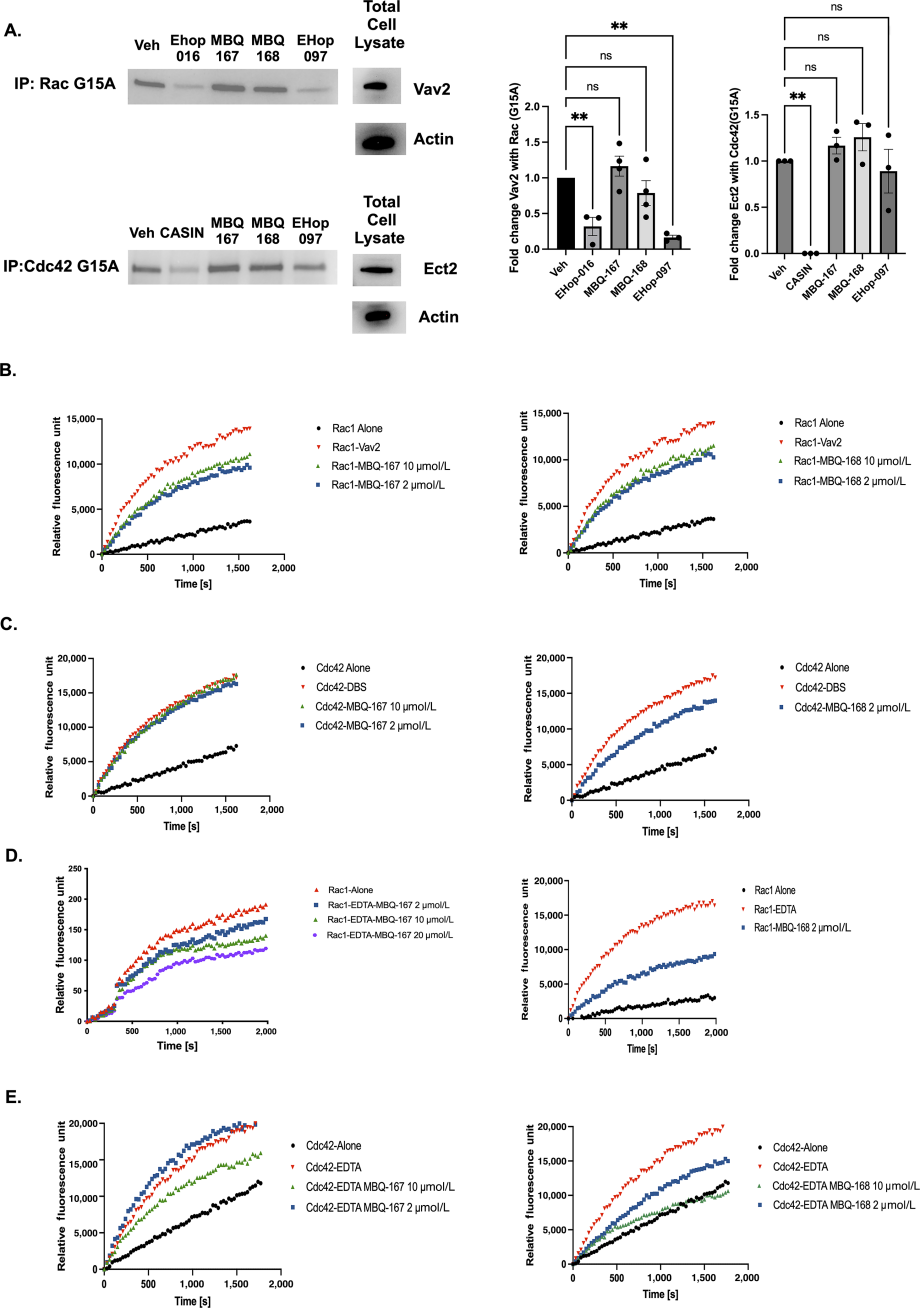
Mechanism of Rac/Cdc42 inhibition by MBQ Compounds. **A,** Rac1 (G15A) or Cdc42 (G15A) coupled to Glutathione sepharose beads were incubated with vehicle, 8 μmol/L of EHop-016, 10 μmol/L of CASIN or 250 nmol/L of MBQ-167, MBQ-168, or EHop-097 for 1 hour and incubated with MDA-MB-231 cell lysates. Left, representative Western blots probed with Vav2, or Ect2 are shown for the Rac1 G15A and Cdc42 G15A pulldowns. Right, Fold change of Vav2 with Rac (G15A) and Ect2 with Cdc42 (G15A). The bar graphs are presented as individual fold change datapoint from vehicle and grouped mean ± SEM (*n* = 3–4; **, *P* < 0.01; ns, not significant). **B,** Nucleotide loading of GTP binding to Rac1 catalyzed by Vav2. Left, Nucleotide loading of N-MAR GTP by Vav2 with MBQ-167; Right, nucleotide loading of N-MAR GTP by Vav2 with MBQ-168. **C,** Nucleotide loading of GTP binding to Cdc42 catalyzed by DBS. Left, nucleotide loading of N-MAR GTP by DBS with MBQ-167; Right, nucleotide loading of N-MAR GTP by DBS with MBQ-168. **D,** Nucleotide loading of GTP binding to Rac1 catalyzed by EDTA. Left, nucleotide loading of Bodipy GTP by EDTA with MBQ-167; Right, Nucleotide loading of N-MAR GTP by EDTA with MBQ-168. **E,** Nucleotide loading of GTP binding to Cdc42 catalyzed by EDTA. Left, Nucleotide loading of N-MAR GTP by EDTA with MBQ-167; Right, nucleotide loading of N-MAR GTP by EDTA with MBQ-168. Rac1 or Cdc42 was incubated with 0 or 2 μmol/L MBQ-167 and the fluorescence of N-MAR-GTP or Bodipy GTP was measured. After initial five readings, Vav2, Dbs, or EDTA at final concentration of 8 mmol/L was added to monitor the loading of N-MAR-GTP or Bodipy GTP to Rac1 or Cdc42.

To identify the GEFs inhibited by MBQ-167 and derivatives, Sepharose-coupled purified Rac1(G15A) or Cdc42(G15A) a mutant form, which interacts tightly with GEFs was incubated with MDA-MB-231 cell lysates to recover GEFs bound to Rac1 or Cdc42, following preincubation with vehicle, or 250 nmol/L MBQ-167, MBQ-168, or EHop-097, as described in ref. 20. MBQ-167 or MBQ-168 had no effect on Rac1(G15A) or Cdc42(G15A) interaction with Vav2 or Ect2 (Fig. 6). Moreover, in this assay, MBQ-167 did not affect Tiam-1, Ect2, or p-REX-1 [data binding to Rac1(G15A; Supplementary Fig. S7)]. Similar to the parent molecule EHop-016 (20–22), Ehop-097 blocked the interaction of Rac1(G15A) with Vav2, albeit at 4X more efficacy at 250 nmol/L, compared with EHop-016 at 8 μmol/L, while MBQ-167 and MBQ-168 had no effect on the Vav2/Rac1 interaction (Fig. 6A).

### MBQ-167 and MBQ-168 Inhibit Guanine Nucleotide Association with Rac

To determine the mechanism of Rac1 and Cdc42 activation inhibition by MBQ-167 and MBQ-168, we monitored the loading of N-MAR-GTP to Rac1 in the presence or absence of MBQ-167 or MBQ-168. We recently reported that MBQ-167 acts as a GDP/GTP-binding inhibitor via nuclear magnetic resonance studies with purified Rac1. MBQ-167 addition resulted in a shift of the Glycine12 residue on Rac1, which is critical for interaction with guanine nucleotides (24). When the exchange reaction of Rac1 catalyzed by the GEF Vav2 was investigated, Vav2 increased the GTP loading on Rac1 in contrast to Rac1 alone. At 2 or 10 μmol/L, MBQ-167 or MBQ-168 inhibited GTP loading on Rac1, when catalyzed by Vav2, by at least at 30% (Fig. 6B). When the Rho/Cdc42 exchange factor Dbs (33) was used to catalyze the exchange reaction, MBQ-167 at 2 or 10 μmol/L, did not affect the increased GTP loading on Cdc42 catalyzed by Dbs. However, MBQ-168 at 2 and 10 μmol/L inhibited the exchange reaction catalyzed by Dbs by approximately 10%–20% (Fig. 6C). MBQ-168 at 2 μmol/L did not affect the Dbs-catalyzed exchange of RhoA, thus showing specificity for inhibition of GTP loading on Rac1 and Cdc42 (Supplementary Fig. S8A).

Addition of EDTA can induce guanine nucleotide exchange on small GTPases in the absence of a GEF, as has been shown for the guanine nucleotide exchange inhibitor EHT1864, which is effective at micromolar concentrations (11). Supplementary Figure S8B demonstrates that MBQ-167 is 25× more effective than EHT1864, where 2 μmol/L MBQ-167 resulted in a similar level of inhibition of GTPγS loading on Rac1 bound to Bodipy-tagged GDP, as 50 μmol/L EHT1864. When EDTA was used to catalyze the loading of fluorescently tagged GTP to Rac1, MBQ-167 inhibited the incorporation of Bodipy-GTP on Rac1 in a concentration-dependent manner from 2 to 20 μmol/L with approximately 50% inhibition in the presence of 20 μmol/L MBQ-167. Similarly, MBQ-168 inhibited EDTA-induced loading of N-MAR-GTP on Rac1 by approximately 50% at 2 μmol/L. Therefore, MBQ-168 appears to be more efficient at inhibiting GTP exchange on Rac1 when measured by an *in vitro* exchange assay (Fig. 6D). Similarly, when EDTA was used to induce GTP exchange on Cdc42, MBQ-167 inhibited the nucleotide loading of N-MAR-GTP at 10 μmol/L, while MBQ-168 reduced nucleotide loading at both 2 and 10 μmol/L, where 10 μmol/L MBQ-168 inhibited GTP exchange by 100% (Fig. 6E).

### MBQ Compounds Inhibit Cytochrome P450 Enzymes

The study of the metabolic fate of drugs is an essential and important part of the drug development process, where phase I reactions are mainly carried out by the cytochrome P450 (CYP450), a family of hemeprotein isozymes primarily found in the liver (34). Therefore, we investigated the potential inhibitory effect of MBQ-167 and structural analogs on the isozymes CYP3A4, CYP2C9, CYP2C19, and CYP1A2 that are among the principal CYP450 enzymes responsible for drug metabolism (35).

**FIGURE 7:**
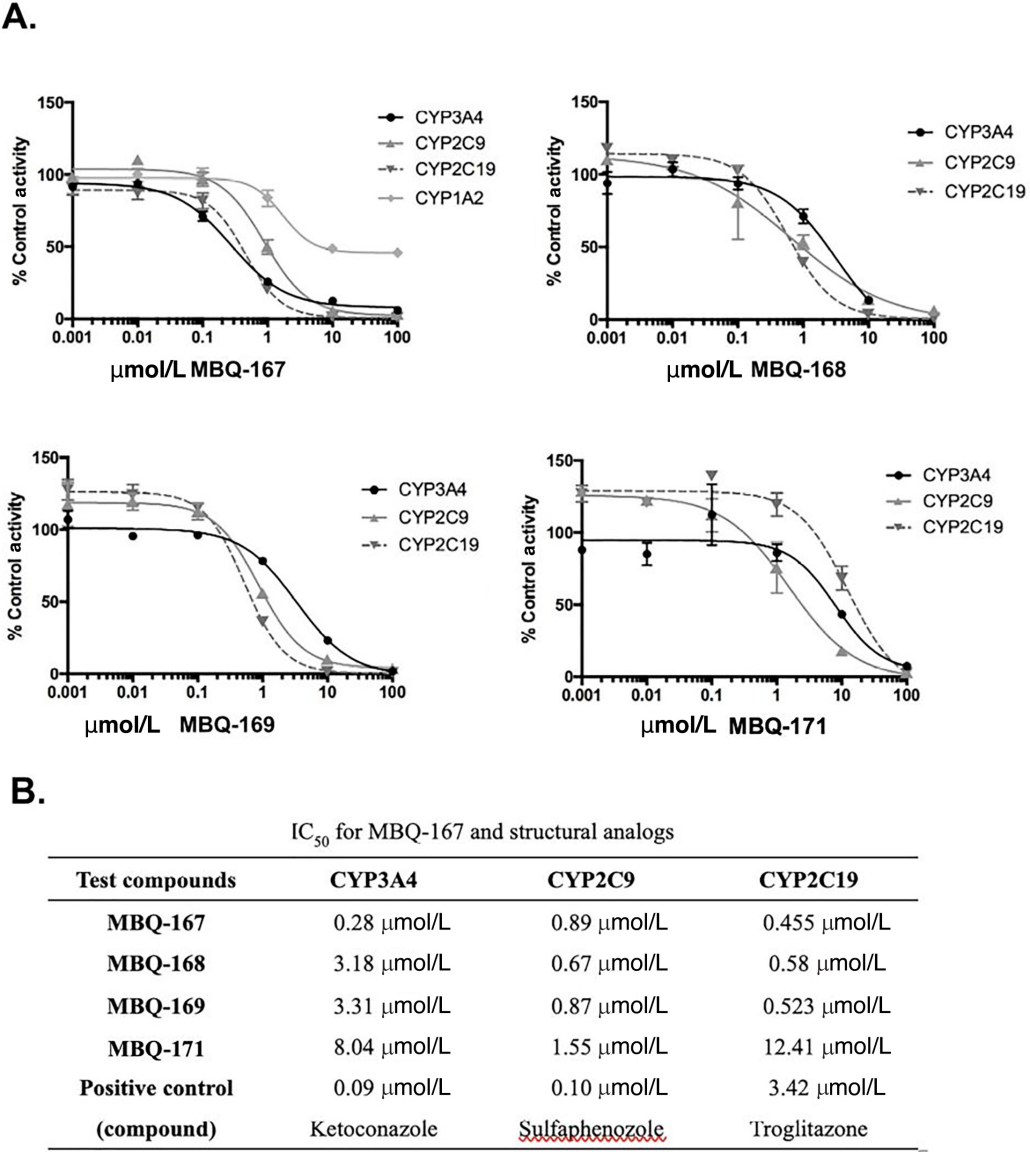
CYP450 enzyme inhibition by MBQ-167 and derivatives. MBQ compounds at 0.001–100 μmol/L were incubated for 10 minutes at room temperature with the reaction mixture of each CYP enzyme, substrate, and buffer. NADPH regeneration system was added to initiate the reaction and incubated for 30 minutes. Next, Luciferin was added for 20 minutes and the chemiluminescence quantified in a luminometer. **A,** Percentage activity following MBQ-167, -168, -169, or -171 relative to vehicle control (100%) is shown for CYP3A4, CYP2C9, and CYP2C19. **B,** IC_50_s for CYP450 enzyme inhibition for each MBQ compound compared with positive control.

The potential for MBQ-167, MBQ-168, MBQ-169, and MBQ-171 to inhibit CYP3A4, CYP2C9, CYP1A2, and CYP2C19 activities was determined using an *in vitro* enzyme assay. The strongest inhibitory activity of MBQ-167 was seen on CYP3A4, followed by CYP2C19 > CYP2C9 > CYP1A2 (Fig. 7A). Greatest inhibition of CYP3A4 activity was seen with MBQ-167 (IC_50_ = 0.28 μmol/L), followed by MBQ-168 (IC_50_ = 3.18 μmol/L) > MBQ-169 (IC_50_ = 3.31 μmol/L) > MBQ-171 (IC_50_ = 8.04 μmol/L) > (IC_50_ = 15.03 μmol/L). MBQ-167 was also the most potent inhibitor of CYP2C19 with an IC_50_ of 0.455 μmol/L. This IC_50_ is even lower than that obtained with the positive control troglitazone (IC_50_ = 3.42 μmol/L). MBQ-168 and MBQ-169 also demonstrated IC_50_s lower than the positive control, with IC_50_s of 0.523 μmol/L and 0.58 μmol/L, respectively. Hence, these are strong CYP2C19 inhibitors. In terms of CYP2C9, the most potent inhibitor was MBQ-168 (IC_50_ = 0.67 μmol/L), followed by MBQ-169 (IC_50_ = 0.87 μmol/L) > MBQ-167 (IC_50_ = 0.89 μmol/L) > MBQ-171 (IC_50_ = 1.55 μmol/L) > (IC_50_ = 8.48 μmol/L; Fig. 7B). Therefore, the compounds that demonstrated potent inhibitory activity against Rac and Cdc42 activation, MBQ-167 and MBQ-168 are also CYP450 inhibitors and this property will need to be considered in drug interactions.

## Discussion

Several inhibitors for Rac1 and Cdc42 activation are available, but the effective concentrations of the reported compounds are too high to be therapeutically useful (7, 8). Our lead compound MBQ-167 is more effective and is in a more advanced stage of development as a metastatic cancer therapeutic, compared with most other available inhibitors. MBQ-167 is effective at nmol/L concentrations as a Rac/Cdc42 inhibitor, and blocks metastasis in several mouse models (23–25, 36–38). Moreover, GLP toxicity studies in rats and dogs have demonstrated safety of MBQ-167 with a NOAEL of 1,000 mg/kg. Therefore, the FDA recently granted Investigational New Drug status for MBQ-167, and we are poised to conduct phase I clinical trials in patients with TNBC. This article describes the characterization of a panel of MBQ-167 derivatives with the goal of elucidating different activities in different cancer cell types where distinct Rac/Cdc42 GEFs are active.

The Rac/Cdc42 downstream effector group I PAKs (PAKs 1–3) are direct downstream effectors of Rac and Cdc42 that have been the subject of intense investigation for targeted anticancer drugs due to their central role in cancer progression to metastasis (39–42). However, drugging the serine and/or threonine kinases PAK1–3 with ATP competitive inhibitors, such as FRAX1037, had limited success in clinical trials due to their poor target selectivity and thus, toxicity. Our data demonstrating that MBQ-168 is more effective at inhibiting both residues of major phosphorylation and/or activation sites, Thr 423 and Ser 141,144,154 in group 1 PAKs, signifies a more efficient mode of inhibition by MBQ-168 or direct inhibition of PAK serine phosphorylation. Therefore, MBQ-168 has potential as a novel Rac/Cdc42 inhibitor with potent anti-PAK activity.

The EHop-016 derivative EHop-097, a 1,4,5-trisubstituted-1,2,3-triazole derivatives was also selected as a potential compound for further analysis because this compound decreased MDA-MB-231 and GFP-HER2-BM cell viability and increased apoptosis. However, for EHop-097, the GI_50_ for the transformed mammary epithelial cell line MCF10A was similar to the approximately 300 nmol/L GI_50_ in breast cancer cells. In contrast, MBQ-168 exhibited a higher GI_50_ in the MCF10A cell line, much as MBQ-167, achieving a low toxicity for this immortalized mammary epithelial cell line. In the ER/PR-positive MCF-7 cells, MBQ-168 demonstrated a lower GI_50_ compared with MBQ-167, therefore having a higher cytotoxicity effect in this cell line, which has more epithelial characteristics. Moreover, the primary human mammary epithelial cell line HMEC, was not inhibited by MBQ-167, MBQ-168, or EHop-097, thus demonstrating nontoxic effects of these compounds in nonimmortalized epithelial cells.

Studies on the effect of MBQ-167, MBQ-168, and EHop-097 on cancer cell migration and actin cytoskeletal extensions show that all three compounds reduce cell migration to a similar extent. The observation that MBQ-168 exhibit an antimigratory effect as MBQ-167, with decreased actin-based ruffles and migratory extensions in both breast and lung cancer cells combined with a loss in cell polarity and ultimate detachment from the substratum to result in cell death (anoikis), demonstrates that MBQ-168 is acting in a similar mechanism as MBQ-167. However, we found enhanced inhibitory performance for MBQ-168 in reducing actin cytoskeletal extensions in response to EGF in lung cancer cells. Therefore, MBQ-168 may be a more effective inhibitor of lung cancer progression than MBQ-167, which will be tested in future preclinical studies using lung cancer mouse models. In contrast, EHop-097 reduced actin cytoskeletal extensions without affecting cell polarity and was less effective at inhibition of EGF-induced actin ruffles in a lung adenocarcinoma cell line; demonstrating a different mechanism of action, as would be predicted by the structural and mechanistic differences of the compounds, as well as the different compliment of exchange factors that are active in lung cancer cells. For instance, it was recently reported that the A549 lung cancer cell line was dependent on the GEFs ARHGEF39, FARP-1, and TIAM-2 while the MDA-MB-231 and GFP-HER2-BM cells have a different GEF compliment and express Vav (29). Therefore, the Vav/Rac inhibitor EHop-097 is expected to be less effective in lung cancer cells.

Similar to the effects on breast cancer cell viability and apoptosis, MBQ-168 inhibited tumor growth to the same extent as MBQ-167 in mice bearing GFP-HER2-BM mammary tumors. This drastic reduction in primary tumors was also reflected in reduced lung, liver, and kidney metastases to a similar extent by MBQ-167 and MBQ-168 demonstrating the feasibility of developing MBQ-168 as an additional anticancer compound. Future studies will determine the bioavailability and toxicity and/or safety of MBQ-168, as well as its effect in a range of cancer cell lines, for its continued translational development.

Rac, Cdc42, and Rho activation assays conducted from cells preincubated with MBQ-167, MBQ-168, or EHop-097, demonstrated that these compounds were selective Rac/Cdc42 inhibitors without affecting Rho activation. MBQ-167 and MBQ-168 had similar inhibitory effects on Rac/Cdc42 activation. Total Rac1 levels were also reduced in tumors from mice that received MBQ-167 or MBQ-168 (5× a week for 2 months), indicating that prolonged treatment of MBQ compounds may affect Rac1 expression at the transcriptional or translational level. Protein stability is also a potential regulatory mechanism because Rac is also known to be ubiquitinated and degraded (43). We also found that in the MDA-MB-468 cell line, which expresses the Rac1B splice variant (32), MBQ-167, MBQ-168, and EHop-097 inhibited activation of Rac1B. This is relevant because Rac1B exhibits accelerated GDP/GTP exchange and an impaired GTP hydrolysis compared with Rac1 (31). Therefore, Rac1B is thought to be oncogenic, even though an antagonistic role for Rac1B in TGFβ signaling has also been reported (32, 44, 45). We found MBQ-167 and EHop-097 to be more effective than MBQ-168 at Rac1B inhibition, indicating mechanistic differences between MBQ-167 and MBQ-168 in blocking guanine nucleotide incorporation. In addition, MBQ-167 also inhibits the (P29S) oncogenic Rac1 mutation expressed in the MDA-MB-157 cell line (46–47). Therefore, these Rac/Cdc42 inhibitors have the potential to also inhibit oncogenic fast cycling mutations of Rac1, in addition to the wildtype Rac1,2,3 isoforms.

By monitoring the nucleotide loading of N-MAR GTP, we found that MBQ-168 was able to block GTP loading when catalyzed by the GEFs Vav2, Dbs or by intrinsic exchange using EDTA. Because we demonstrated that our compounds do not block the GEFs Vav2, Ect2, p-REX, Tiam1, etc., it can be construed that MBQ-167 and MBQ-168 impede nucleotide loading independent of the GEFs by binding the G12 residue and not a specific GEF-interacting site, as was shown previously for inhibition of the Vav/Rac interaction for EHop-016 (20), and in this study, for EHop-097. Because we investigated only a subset of potential GEFs, MBQ-167 or MBQ-168 may block a specific GEF yet to be identified.

As would be predicted by the action of an established guanine nucleotide association inhibitor (11), both MBQ-167 and MBQ-168, inhibited the exchange activity induced by the Ca^2+^/Mg^2+^ Chelator EDTA. The observed enhanced inhibition of EDTA-induced GTP loading on both Rac and Cdc42 by MBQ-168 compared with MBQ-167 demonstrates that MBQ-168 is more effective than MBQ-167. This superior efficacy of MBQ-168 may be attributed to MBQ-167’s poor solubility, pH sensitivity, or fluorescence interference due to the intrinsic fluorescence spectra of MBQ-167 rather than diminished efficacy because both MBQ-167 and MBQ-168 inhibit Rac activation from breast cancer cell lysates to a similar extent.

MBQ-167 inhibits Cyp450 enzyme CYP3A4 activity but to a lesser extent than the positive control, which has an IC_50_ that is 3-fold lower at the same concentration, while the other derivatives MBQ-168, -169, and -171 had higher IC_50_s. MBQ-168 was found to be approximately 10X less potent than MBQ-167 at inhibiting CYP3A4. This observation is relevant because CYP3A4 availability in tumors may limit the intracellular concentrations of docetaxel and paclitaxel, which are anticancer drugs that are substrates of CYP3A4 (48). Hence, MBQ-167, but not MBQ-168, may potentially inhibit intratumoral metabolism of taxanes, increasing their intracellular concentrations and potentiating their effect as chemotherapeutic agents. Further studies are needed to validate this hypothesis. MBQ-167, -168, and -169 inhibited CYP2C9 but were not as potent as the positive control. However, CYP2C19 was inhibited by MBQ-167, -168, and -169 at IC_50_s that were approximately 6-fold lower than the IC_50_ for the positive control, indicating a potent inhibition of this enzyme. The Ki values for MBQ-167 inhibition of CYP2C9, and CYP2C19 fall within the therapeutic window of MBQ-167 in metastatic breast cancer cells (0.25–0.5 μmol/L; ref. 23). On the basis of these data, the *in vitro* inhibition results indicate that *in vivo*, MBQ-167 and MBQ-168 have the potential to inhibit CYP2C9 and CYP2C19.

In clinical practice, patients with cancer undergo multiple-drug therapy. The concomitant use of MBQ-167 and analogs with clinically prescribed drugs that are substrates of CYP3A4, CYP2C9, and CYP2C19 isoforms could cause potential drug–drug interactions. Inhibition of CYP enzymes *in vivo* may result in unexpected elevations in the plasma concentrations of concomitant drugs, leading to adverse effects (49). Hence, if MBQ-167 or analogs are approved for clinical use, dose adjustments and timing of administration might be necessary to prevent adverse effects and toxicity in the clinic.

In conclusion, this study highlights the importance of developing structural analogs of our previously characterized Rac/Cdc42 inhibitors EHop-016 and MBQ-167. While MBQ-167 is being developed as our lead antimetastatic cancer compound, the panel of derivatives that are being characterized can be used for different applications in different cancer types. The efficacy of MBQ-167 and MBQ-168 in both metastatic breast and lung cancer cell lines reinforce the mechanism of action described here, where the MBQ compounds inhibit GTP binding and not GDP/GTP exchange by GEFs. Therefore, MBQ-167 and MBQ-168 can be used to inhibit Rac/Cdc42 in multiple cancers regardless of the individual oncogenic GEF activity. However, MBQ-168 is more soluble than MBQ-167 due to the methoxy group, which can facilitate polar contact and is therefore, is predicted to be more bioavailable.

This salient observation that EHop-097 is a Vav/Rac specific inhibitor demonstrates its utility in inhibiting specific cancer cells, as well as immune cells, where the Vav1/Rac2 activation is central to immune cell function (50). Therefore, the mechanism of action of EHop-097is distinct from MBQ-167 and MBQ-168, and is similar to EHop-016, and is predicted to act as a competitive inhibitor at the Vav/Rac-binding site. However, EHop-097 is approximately 10–30× more potent than EHop-016. Therefore, EHop-097 can be a specific and potent inhibitor in cancer cell types and immune cells where the oncogene Vav is the major regulator of Rac signaling.

## Supplementary Material

Supplementary Methods SM1 Supplementary methods

Suppl. Fig. S1 Suppl. Fig. S1 shows percentage cell viability

Suppl. Fig. S2 Supplemental Figure S2 shows the Effect of MBQ compounds on cell migration

Suppl. Fig. S3 Suppl. Fig. S3 shows the effect of MBQ-167 and derivatives on lung cancer cells.

Suppl. Fig. S4 Supplementary Figure S4 shows the safety of MBQ compounds in mice.

Suppl. Fig. S5 Supplementary Figure S5 shows the effect of MBQ compounds on Rac activation in attached and detached cells.

Suppl. Fig. S6 Supplementary Figure S6 shows the inhibition of Rac1B, Rac1, and Rac1 P29S by MBQ-167

Suppl. Fig. S7 Supplementary Figure S7 shows the effect of MBQ-167 on Rac1(G15A) association with Tiam-1 and p-REX-1.

Suppl. Fig. S8 Supplementary Figure S8 shows nucleotide loading of GTP binding to Rho GTPases.
