# Supplementary material for: Characterization of Novel Derivatives of MBQ-167, an Inhibitor of the GTP-binding Proteins Rac/Cdc42
Source: Cancer Res Commun. 2022 Dec 29;2(12):1711–26. doi: 10.1158/2767-9764.CRC-22-0303 (PMC9970268; doi:10.1158/2767-9764.CRC-22-0303)
Supplement: Supplementary Methods SM1 — Supplementary methods [file crc-22-0303-s01.pdf]

Supplementary Materials and Methods

Synthetic methods

General procedures

All experiments were carried out in pre-dried glassware (≥1 h, 80-90°C) under a nitrogen atmosphere. The progress of the reaction was monitored via TLC (Silica gel with fluorescent indicator and 60 Å medium pore diameter) from Sigma-Aldrich.. Nuclear magnetic resonance (NMR) spectra were obtained using a 400 MHz or a 500 MHz Bruker Avance UltraShield™ spectrometer. ^{1}H (400/500 MHz) and ^{13}C (100/125 MHz) NMR were recorded in $CDCl_3$ or DMSO-d_6, unless otherwise noted, and the chemical shifts were expressed in ppm with $CDCl_3$ (δ 7.26 ppm for ^{1}H and δ 77.0 ppm for ^{13}C) or DMSO-d_6 (δ 2.50 ppm for ^{1}H and δ 39.5 ppm for ^{13}C) as internal standard. Low resolution gas chromatography mass spectrometry (LR-GC MS) spectral data were obtained with a GC Agilent 6890N spectrometer (70 eV) and a 5973 Agilent Inert Mass Selective Detector.. Electrospray ionization Fourier transform mass spectrometry (FTMS-ESI) data were obtained with an Agilent 1200 Series Gradient HPLC System with an autosampler temperature of 15 ºC and coupled to a Thermo Fisher Q-Exactive Orbitrap Mass Spectrometer. The synthesis of 3-azido-9-ethyl-9*H*-carbazole (Azide, **3**) was performed as described in (14). To avoid any potential hazards to personnel or environment, and to ensure safety of all individuals working in the laboratory, special precautions were exercised during the experimental procedures, and the synthesis was carried out in a well-ventilated hood due to the formation of extremely toxic and potentially explosive hydrazoic acid.

General procedure for the synthesis of 5-aryl modified derivatives of MBQ-167 (1a-e).

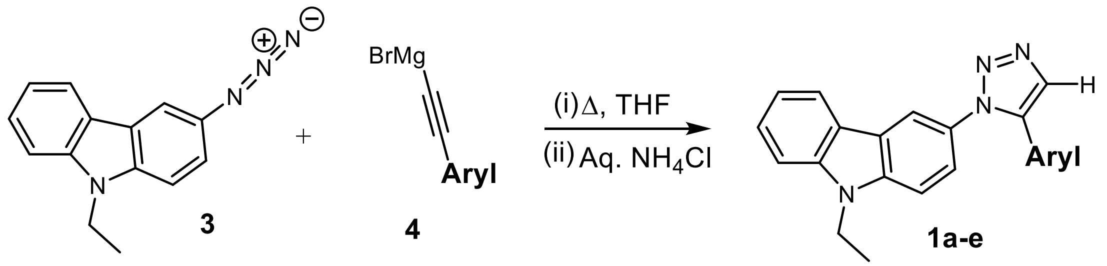

Scheme 1. Synthesis of 5-aryl-modified derivatives of MBQ-167; reagents and conditions; (i) THF, 50ºC, 1 h; (ii) NH_4Cl (aq).

In a 25-mL three-neck round-bottom flask containing the terminal alkyne (1.0 mmol) under a nitrogen atmosphere, a solution of 1.0 M ethylmagnesium bromide in THF (1.1 mmol) was added drop-wise at 25 ºC. After the Grignard reagent was added, the mixture was heated at 50 ºC for 15 minutes and cooled to 25 ºC. A solution of 0.24 g (1.0 mmol) of azide **3** in THF (1.0 mol/L) was added drop-wise and heated to 50 ºC for 1 hour. After quenching with 10% NH_4Cl, the products were extracted with ethyl acetate (3x). The combined organic fractions wer washed with 10 mL brine, separated and dried on sodium sulfate, filtered and concentrated on a rotary evaporator to obtain the crude material. The crude oil was purified by silica gel flash column chromatography to afford the corresponding 1,5-disubstituted-1,2,3-triazole derivative.

9-ethyl-3-(5-(3-methoxyphenyl)-1H-1,2,3-triazol-1-yl)-9H-carbazole (**1a**, MBQ-168)

Light-brown solid (0.74 mmol, 74%, 0.2727 g); R_f = 0.43 (Hexane/Ethyl Acetate, 3:1); ^{1}H NMR ($CDCl_3$, 400 MHz) δ 1.48 (t, 3H, *J* = 7.23 Hz), 3.91 (s, 3H), 4.43 (q, 2H, *J* = 7.24 Hz), 6.93 (d, 1H, *J* = 8.20 Hz), 7.30 (t, 1H, *J* = 7.77 Hz), 7.38 (t, 1H, *J* = 7.95 Hz), 7.48 (s, 1H), 7.51 (d, 1H, *J* = 8.70 Hz), 7.56 (d, 1H, *J* = 12.22 Hz), 7.84 (d, 1H, *J* = 2.14 Hz), 7.86 (d, 1H, *J* = 2.12 Hz), 8.15 (d, 1H, *J* = 7.75 Hz), 8.25 (s, 1H), 8.44 (s, 1H) ppm; ^{13}C NMR ($CDCl_3$, 100 MHz) δ 13.82, 37.87, 55.41, 108.99, 109.08, 110.91, 113.30, 114.42, 118.26, 118.64, 119.03, 119.56, 120.84, 122.56, 123.37, 126.74, 129.47, 129.93, 131.92, 139.64, 140.80, 148.09, 160.13 ppm. LR-GC MS *m/z* (rel%): $[M]^+$ 368 (13), $[M-N_2]^+$ 340 (69), $[M-CH_3N_2]^+$ 325 (35), $[M-C_{11}H_{13}O]^+$ 207 (100).

9-ethyl-3-(5-(m-tolyl)-1H-1,2,3-triazol-1-yl)-9H-carbazole, (**1b**, MBQ-169)
Bone-white solid (0.40 mmol, 40%, 0.1418 g); R_f = 0.28 (Hexane/Ethyl Acetate, 3:1); ^{1}H NMR ($CDCl_3$, 400 MHz) δ 1.45 (t, 3H, *J* = 7.24 Hz), 2.27 (s, 3H), 4.38 (q, 2H, *J* = 7.24 Hz), 6.99 (d, 1H, *J* = 1.37 Hz), 7.14 (t, 1H, *J* = 6.96 Hz), 7.25 (t, 1H, *J* = 6.94 Hz), 7.35 (d, 1H, *J* = 8.64 Hz), 7.39 (d, 1H, *J* = 8.62 Hz), 7.44 (d, 1H, *J* = 8.22 Hz), 7.50 (d, 1H, *J* = 7.62 Hz), 7.53 (s, 1H), 7.91 (s, 1H), 8.02 (d, 1H, *J* = 7.78 Hz), 8.17 (s, 1H) ppm; ^{13}C NMR ($CDCl_3$, 100 MHz) δ 14.04, 21.60, 38.08, 108.90, 109.19, 118.05, 119.76, 121.06, 122.79, 123.25, 123.42, 125.88, 126.86, 127.24, 128.68, 128.85, 129.45, 130.01, 133.347, 138.40, 138.81, 139.99, 140.92 ppm. LR-GC MS *m/z* (rel%): $[M]^+$ 352 (31), $[M-N_2]^+$ 324 (67), $[M-C_3H_8]^+$ 309 (100), $[M-C_{11}H_{13}N_2]^+$ 179 (42).

9-ethyl-3-(5-(pyridin-2-yl)-1H-1,2,3-triazol-1-yl)-9H-carbazole, (**1c**, MBQ-170).
Light-brown solid (0.66 mmol, 66%, 0.224 g); R_f = 0.44 (Hexane/Ethyl Acetate, 3:1); ^{1}H NMR ($CDCl_3$, 400 MHz) δ 1.48 (t, 3H, *J* = 7.13 Hz), 4.43 (q, 2H, *J* = 7.26 Hz), 7.29 (t, 1H, *J* = 13.54 Hz), 7.47 (d, 1H, *J* = 8.07 Hz), 7.53 (t, 1H, *J* = 7.77 Hz), 7.83 (t, 1H, *J* = 7.81 Hz), 7.87 (d, 1H, *J* = 2.11 Hz), 7.89 (d, 1H, *J* = 2.13 Hz), 8.13 (d, 1H, *J* = 7.36 Hz), 8.29 (d, 1H, *J* = 7.19 Hz), 8.49 (s, 1H), 8.64 (d, 1H, *J* = 4.05 Hz), 8.68 (s, 1H) ppm; ^{13}C NMR ($CDCl_3$, 100 MHz,) δ 13.82, 37.86, 108.97, 109.13, 113.17, 118.76, 119.59, 120.424, 120.66, 120.83, 122.56, 122.95, 123.38, 126.74, 129.39, 136.98, 139.65, 140.78, 148.765, 149.48, 150.30 ppm. LR-GC MS *m/z* (rel%): $[M]^+$ 339 (9), $[M-N_2]^+$ 311 (32), $[M-C_2H_5N]^+$ 296 (100), $[M-C_9H_{10}N_3]^+$ 179 (18).

9-ethyl-3-(5-(pyridin-3-yl)-1H-1,2,3-triazol-1-yl)-9H-carbazole, (**1d**, MBQ-171)
Light-brown solid (0.31 mmol, 31%, 0.1035 g); R_f = 0.20 (Hexane/Ethyl Acetate, 3:1); ^{1}H NMR ($CDCl_3$, 400 MHz) δ 1.47 (t, 3H, *J* = 7.18 Hz), 4.39 (q, 2H, *J* = 7.14 Hz), 7.22 (d, 1H, *J* = 3.03 Hz), 7.26 (t, 1H, *J* = 7.39 Hz), 7.34 (d, 1H, *J* = 1.82 Hz), 7.36 (d, 1H, *J* = 1.79 Hz), 7.42 (s, 1H), 7.45 (d, 1H, *J* = 4.37 Hz), 7.52 (t, 1H, *J* = 7.35 Hz), 8.01 (d, 1H, *J* = 5.98 Hz), 8.12 (s, 1H), 8.56 (d, 1H, *J* = 4.64 Hz), 8.62 (s, 1H); ^{13}C NMR ($CDCl_3$, 100 MHz,) δ 13.72, 37.75, 108.92, 117.80, 119.57, 120.69, 122.20, 122.37, 122.17, 123.23, 123.35, 123.40, 126.73, 127.49, 133.20, 134.95, 135.48, 139.82, 140.61, 148.88, 149.88. LR-GC MS *m/z* (rel%): $[M]^+$ 339 (34), $[M-N_2]^+$ 311 (42), $[M-C_2H_5N]^+$ 296 (100), $[M-C_2H_5N_2]^+$ 281 (76), $[M-C_9H_{10}N_3]^+$ 179 (37).

9-ethyl-3-(5-(thiophen-3-yl)-1H-1,2,3-triazol-1-yl)-9H-carbazole, (**1e**, MBQ-172)
Brown solid (0.34 mmol, 34%, 0.1179 g); R_f = 0.54 (Hexane/Ethyl Acetate, 1:1); ^{1}H NMR ($CDCl_3$, 400 MHz) δ 1.50 (t, 3H, *J* = 7.24 Hz), 4.43 (q, 2H, *J* = 7.24 Hz), 6.97 (d, 1H, *J* = 5.04 Hz), 7.09 (d, 1H, *J* = 2.84 Hz), 7.27 (t, 1H, *J* = 2.96 Hz), 7.45 (d, 1H, *J* = 1.90 Hz), 7.48 (d, 1H, *J* = 6.14 Hz), 7.53 (d, 1H, *J* = 7.20 Hz), 7.95 (s, 1H), 8.04 (d, 1H, *J* = 7.79 Hz), 8.16 (s, 1H) ppm; ^{13}C NMR (CDCl3, 100 MHz,) δ 13.84, 37.91, 108.85, 108.98, 118.27, 119.65, 120.85, 122.46,

123.25, 123.31, 124.34, 126.48, 126.75, 126.94, 127.04, 128.24, 132.31, 134.11, 140.04, 140.73 ppm. LR-GC MS *m/z* (rel%): $[M]^+$ 344 (39), $[M-N_2]^+$ 316 (25), $[M-C_2H_5N]^+$ 301 (49), $[M-C_8H_9S]^+$ 207 (100), $[M-C_8H_9N_2S]^+$ 179 (36).

General procedure for the synthesis of 4-(aryl)methanol derivatives of MBQ-167 (2a-c)

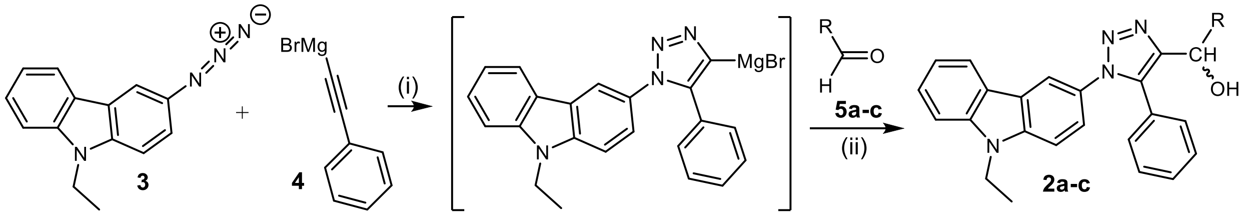

Scheme 2. Synthesis of 4-(aryl)methanol derivatives of MBQ-167; reagents and conditions; (i) THF, 50ºC, 1 h; (ii) (a) RCHO (R = aryl or heteroaryl ring), 1 h, room temperature, (b) NH_4Cl (aq)

In a 25-mL three-neck round-bottom flask containing phenylacetylene (1.0 mmol) under a nitrogen atmosphere, a solution of 1.0 M ethylmagnesium bromide in THF (1.1 mmol) was added drop-wise at 25 ºC. After the Grignard reagent was added, the mixture was heated at 50 ºC for 15 minutes and cooled to 25 ºC. Neat azide (**3**) (0.8 mmol) was added and the reaction mixture heated to 50 ºC for 1 hour. After addition of the corresponding aldehyde **5a-c** (1.0 mmol, neat) and stirring for 1 hour at room temperature, a solution of 10% ammonium chloride was added and the products extracted with ethyl acetate (3x). The combined organic fractions were washed with brine (3x), separated and dried on sodium sulfate, filtered and concentrated on a rotary evaporator to obtain the crude material. The crude oil was purified by silica gel flash column chromatography to afford the corresponding 1,4,5-trisubstituted-1,2,3-triazole derivative.

(1-(9-ethyl-9H-carbazol-3-yl)-5-phenyl-1H-1,2,3-triazol-4-yl)(furan-2-yl)methanol (**2a**, EHop-098).
Brown solid (0.1 mmol, 13%, 0.0437 g); R_f = 0.52 (Hexane/Ethyl Acetate, 1:1); ^{1}H-NMR ($CDCl_3$, 500 MHz) δ 1.46 (t, *J* = 7.2 Hz, 3H), 1.59 (s, 1H), 2.06 (s, 1H), 4.39 (q, *J* = 7.2 Hz, 2H), 5.93 (d, *J* = 6.4 Hz, 1H), 6.44 – 6.35 (m, 5H), 7.40 – 7.16 (m, 5H), 7.44 (dd, *J* = 8.3, 4.6 Hz, 1H), 7.52 (dd, *J* = 11.3, 4.1 Hz, 1H), 8.00 (d, *J* = 7.8 Hz, 1H), 8.11 (d, *J* = 1.8 Hz, 1H) ppm; ^{13}C ($CDCl_3$, 100 MHz) δ 13.79, 37.81, 62.74, 108.00, 108.31, 108.56, 108.91, 109.02, 110.44, 117.70, 119.53, 120.85, 122.46, 122.78, 123.06, 126.54, 126.62, 128.07, 128.73, 129.34, 129.82, 139.65, 140.63, 142.57, 154.50 ppm. FTMS (ESI) *m/z* calcd for $C_{27}H_{22}N_4O_2$, $[M+H]^+$ 435.1822, found 435.1796.

(1-(9-ethyl-9H-carbazol-3-yl)-5-phenyl-1H-1,2,3-triazol-4-yl)(pyridin-3-yl)methanol, (**2b**, EHop-040).
Bone-white solid (0.13 mmol, 16 %, 0.0555 g); R_f = 0.26 (Hexane/Ethyl Acetate, 1:1); ^{1}H-NMR ($CDCl_3$, 500 MHz) δ 1.46 (t, *J* = 7.2 Hz, 3H), 2.07 (s, 1H), 4.38 (q, *J* = 7.2 Hz, 2H), 6.06 (s, 1H), 7.18 (d, *J* = 7.1 Hz, 1H), 7.39 – 7.23 (m, 4H), 7.45 (d, *J* = 8.2 Hz, 2H), 7.53 (t, *J* = 7.7 Hz, 2H), 7.96 (dd, *J* = 18.8, 7.9 Hz, 4H), 8.08 (d, *J* = 1.9 Hz, 1H), 8.53 (d, *J* = 4.0 Hz, 1H), 8.62 (s, 1H) ppm; ^{13}C ($CDCl_3$, 100 MHz) δ 13.88, 37.88, 66.76, 108.68, 109.00, 117.70, 119.64, 120.81, 122.50, 122.76, 123.12, 123.56, 126.55, 126.74, 127.99, 128.92, 129.50, 129.96, 135.02, 135.40,

139.74, 140.65, 140,71, 146.24, 148.10, 148.68 ppm. FTMS (ESI) *m/z* calcd for $C_{28}H_{23}N_5O$, $[M+H]^+$ 446.1982, found 446.1954.

(1-(9-ethyl-9H-carbazol-3-yl)-5-phenyl-1H-1,2,3-triazol-4-yl)(3-methoxyphenyl)methanol (**2c**, EHop-097).
Light-brown solid (0.026 mmol, 3.3%, 0.0123 g); R_f = 0.49 (Hexane/Ethyl Acetate, 1:1); ^{1}H-NMR ($CDCl_3$, 500 MHz) δ 1.29 (s, 2H), 1.46 (t, *J* = 7.2 Hz, 3H), 4.38 (q, *J* = 7.2 Hz, 2H), 5.93 (s, 1H), 6.95 – 6.86 (m, 2H), 7.20 – 7.14 (m, 2H), 7.39 – 7.22 (m, 6H), 7.45 (d, *J* = 8.5 Hz, 3H), 7.57 – 7.49 (m, 3H), 7.99 (d, *J* = 7.7 Hz, 1H), 8.22 – 8.06 (m, 1H) ppm; ^{13}C ($CDCl_3$, 100 MHz) δ 13.76, 37.76, 55.27, 68.34, 108.52, 108.86, 113.83, 117.60, 119.46, 120.69, 122.43, 122.73, 122.97, 126.56, 126.83, 128.09, 128.22, 128.61, 129.07, 129.91, 132.22, 134.66, 134.71, 134.77, 139.56, 140.58, 147.09, 159.14 ppm. FTMS (ESI) *m/z* calcd for $C_{30}H_{26}N_4O_2$, $[M+H]^+$ 475.2135, found 475.2106.
