# Supplementary material for: Characterization of Novel Derivatives of MBQ-167, an Inhibitor of the GTP-binding Proteins Rac/Cdc42
Source: Cancer Res Commun. 2022 Dec 29;2(12):1711–26. doi: 10.1158/2767-9764.CRC-22-0303 (PMC9970268; doi:10.1158/2767-9764.CRC-22-0303)
Supplement: Suppl. Fig. S1 — shows percentage cell viability [file crc-22-0303-s02.pdf]

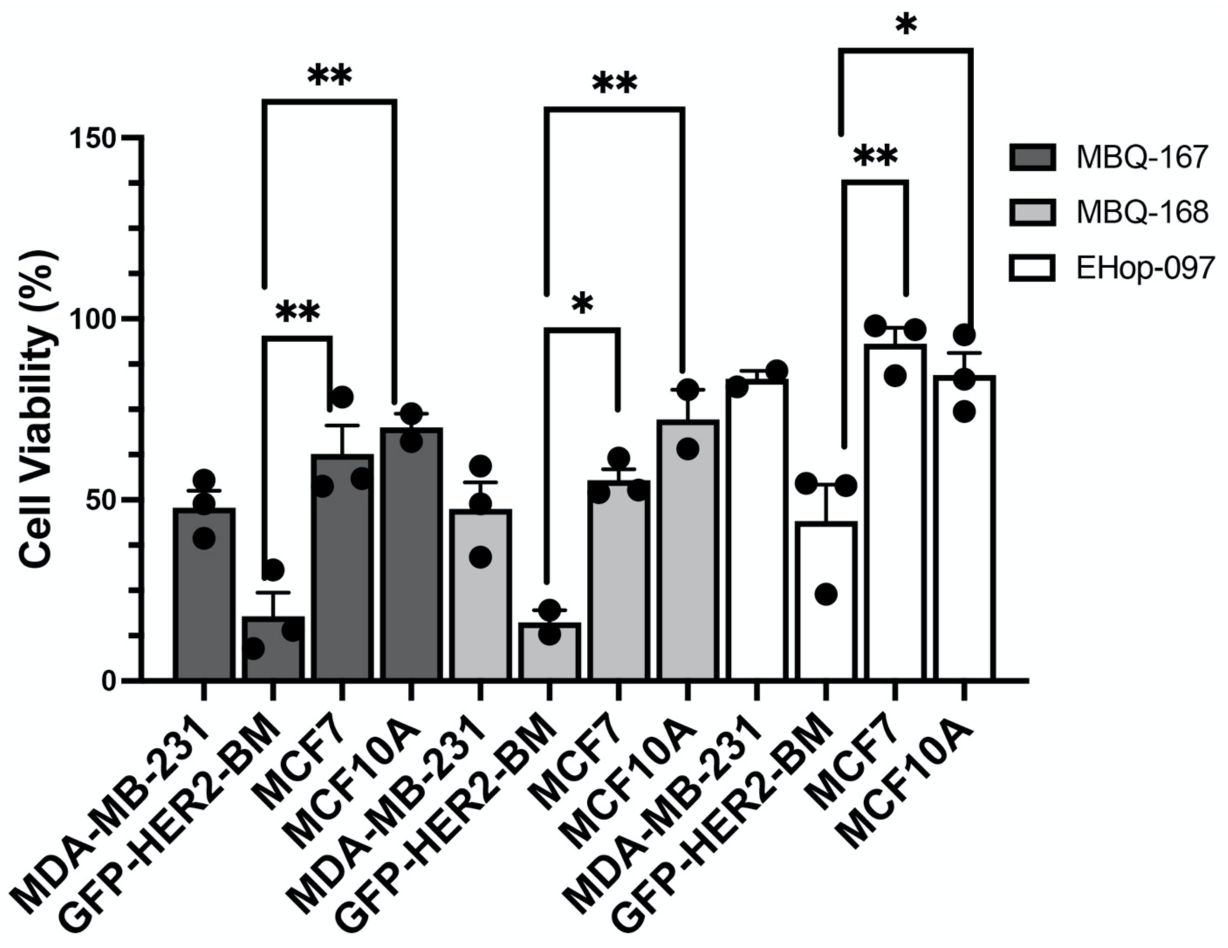

Supplementary Figure S1. Percentage cell viability of MDA-MB-231, GFP-HER2-BM, MCF7 and MCF10A cell lines following MBQ-167, MBQ-168 or Ehop-097 treatment at 250 nM for 120 hr. The bar graphs are presented as individual fold change data points from vehicle and grouped mean ± SEM. (n = 3; *, P < 0.05; ** P < 0.01).
