# Supplementary material for: Characterization of Novel Derivatives of MBQ-167, an Inhibitor of the GTP-binding Proteins Rac/Cdc42
Source: Cancer Res Commun. 2022 Dec 29;2(12):1711–26. doi: 10.1158/2767-9764.CRC-22-0303 (PMC9970268; doi:10.1158/2767-9764.CRC-22-0303)
Supplement: Suppl. Fig. S2 — Supplemental Figure S2 shows the Effect of MBQ compounds on cell migration [file crc-22-0303-s03.pdf]

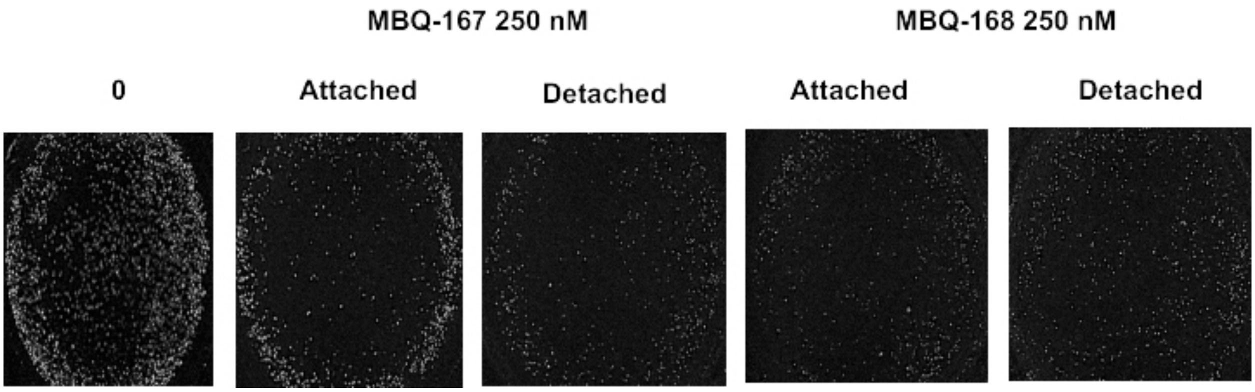

Supplementary Figure S2. Effect of MBQ-167 and MBQ-168 on GFP-HER2-BM cell migration. Cells treated for 24hr and the attached and detached cell populations recovered and equal cell numbers were seeded on Transwells with 8-mm diameter pores and incubated for 7 hr. Cells that migrated to the underside of the membrane were stained with propidium iodide and imaged at 20X. Representatives images are a result from two technical replicates and three biological replicates.
