# Supplementary material for: Characterization of Novel Derivatives of MBQ-167, an Inhibitor of the GTP-binding Proteins Rac/Cdc42
Source: Cancer Res Commun. 2022 Dec 29;2(12):1711–26. doi: 10.1158/2767-9764.CRC-22-0303 (PMC9970268; doi:10.1158/2767-9764.CRC-22-0303)
Supplement: Suppl. Fig. S3 — shows the effect of MBQ-167 and derivatives on lung cancer cells. [file crc-22-0303-s04.pdf]

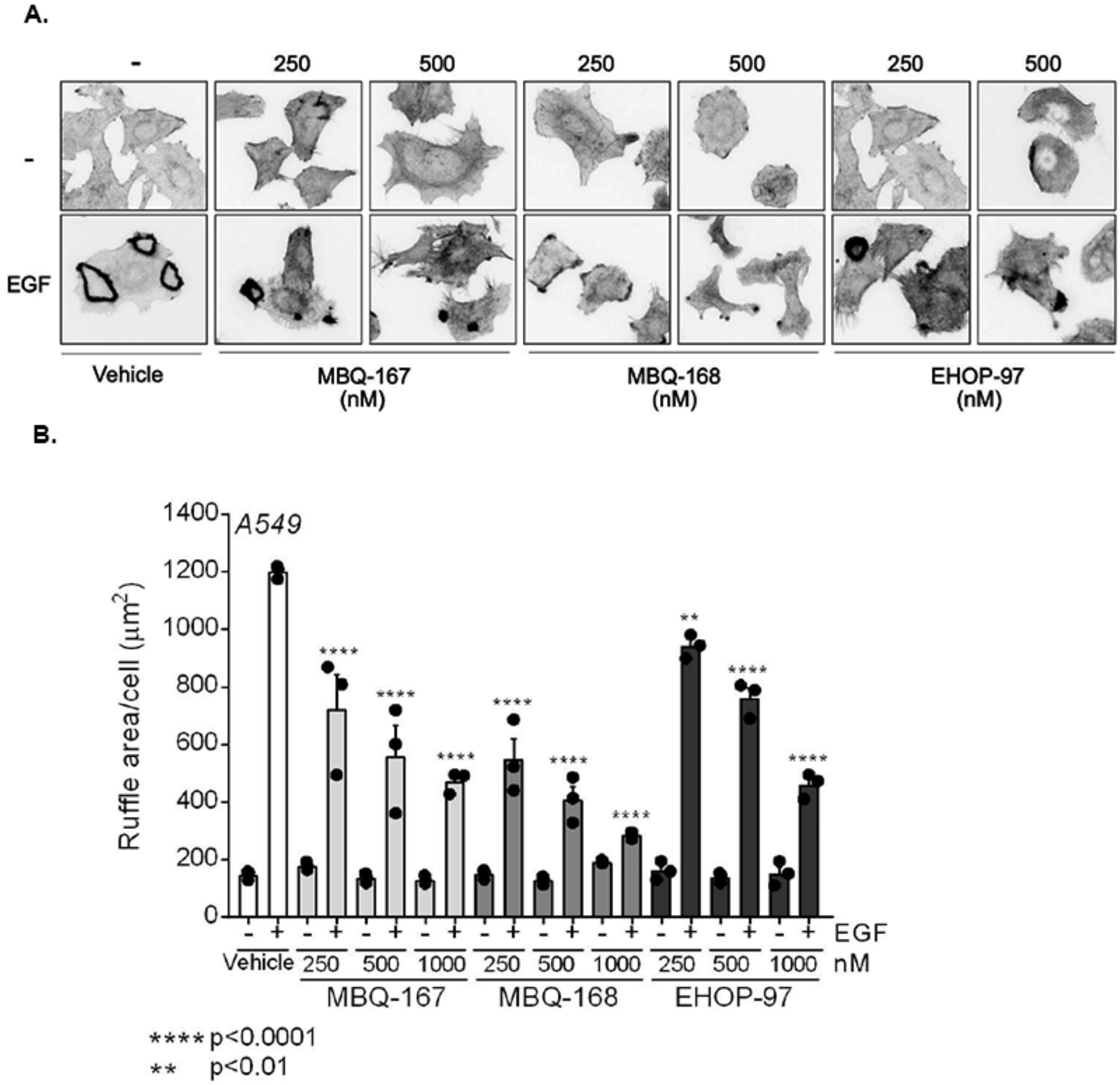

Supplementary Figure S3. Effect of MBQ-167, MBQ-168 and EHop-097 on actin cytoskeleton in A549 cells. Cells were seeded and incubated for 24 hr with 250 or 500 nM of our compounds. Cells were stimulated with EGF at 200 ng/ml and the ruffle area was measured. **A.** Representative images of membrane ruffling after MBQ-167, MBQ-168 and EHop-097 treatments. B. Quantification of ruffle area per cell. (n=3; **, P < 0.01; **** P < 0.0001). Error bars represent SEM.
