# Supplementary material for: Characterization of Novel Derivatives of MBQ-167, an Inhibitor of the GTP-binding Proteins Rac/Cdc42
Source: Cancer Res Commun. 2022 Dec 29;2(12):1711–26. doi: 10.1158/2767-9764.CRC-22-0303 (PMC9970268; doi:10.1158/2767-9764.CRC-22-0303)
Supplement: Suppl. Fig. S4 — Supplementary Figure S4 shows the safety of MBQ compounds in mice. [file crc-22-0303-s05.pdf]

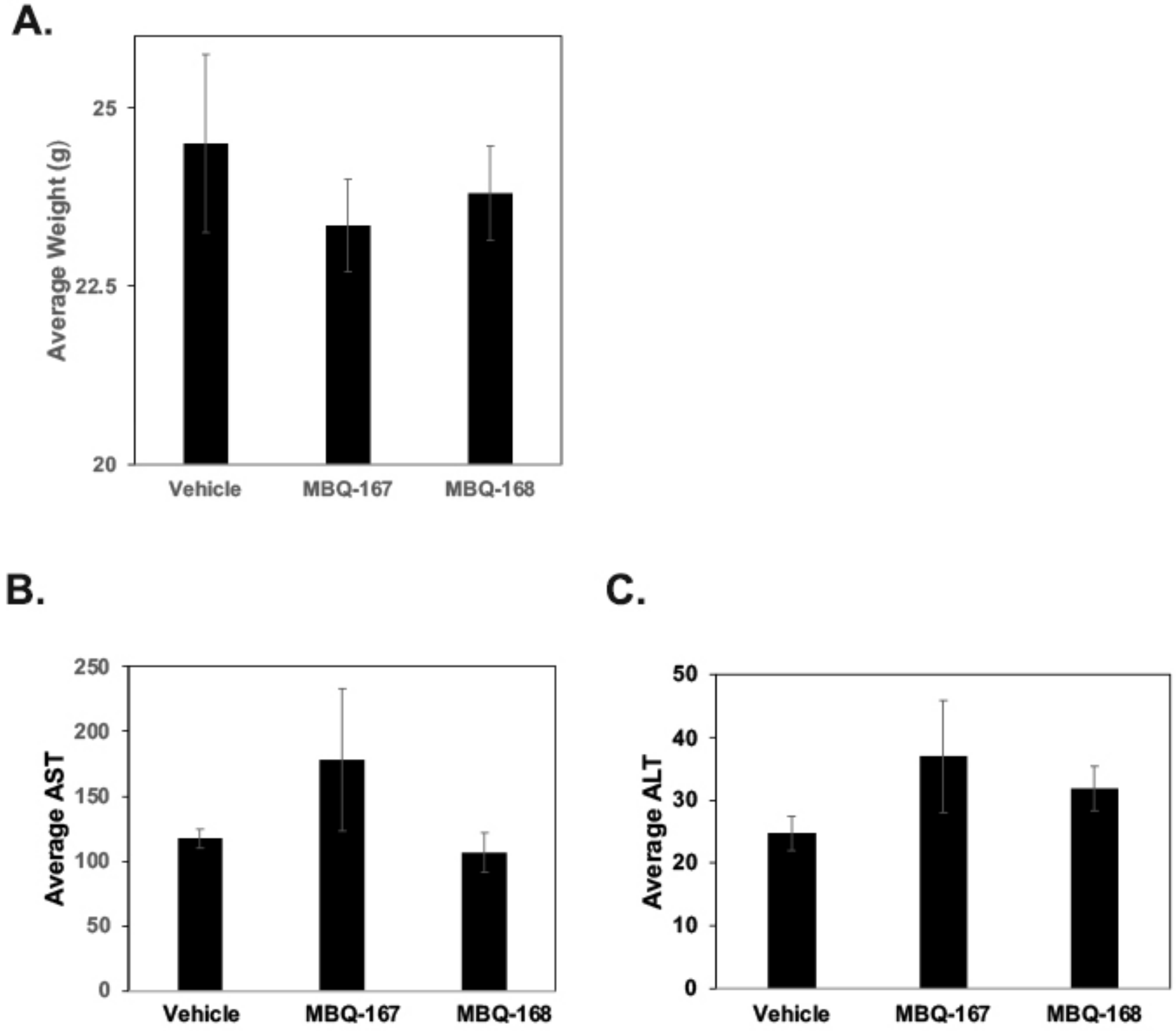


**Supplementary Figure S4**. **MBQ-167 and MBQ-168 effects on toxicity parameters.** SCID mice bearing mammary fatpad tumors from HER2-GFP-BM metastatic cancer cells were treated 5X a week with vehicle, or 5mg/kg MBQ-167 or MBQ-168 5X a week, as described in Figure 3. At the end of the study (in 53 days), mice were weighed, sacrificed, and the plasma (from cardiac puncture) subjected to Aspartate transaminase (AST) or alanine aminotransferase (ALT) levels quantification. **A**. Average mouse weights on day 53. **B**. Average ALT levels. **C**, Average AST levels. N=5±SEM. No significance in Student's T test when treated samples were compared with vehicle.
