# Supplementary material for: Characterization of Novel Derivatives of MBQ-167, an Inhibitor of the GTP-binding Proteins Rac/Cdc42
Source: Cancer Res Commun. 2022 Dec 29;2(12):1711–26. doi: 10.1158/2767-9764.CRC-22-0303 (PMC9970268; doi:10.1158/2767-9764.CRC-22-0303)
Supplement: Suppl. Fig. S5 — Supplementary Figure S5 shows the effect of MBQ compounds on Rac activation in attached and detached cells. [file crc-22-0303-s06.pdf]

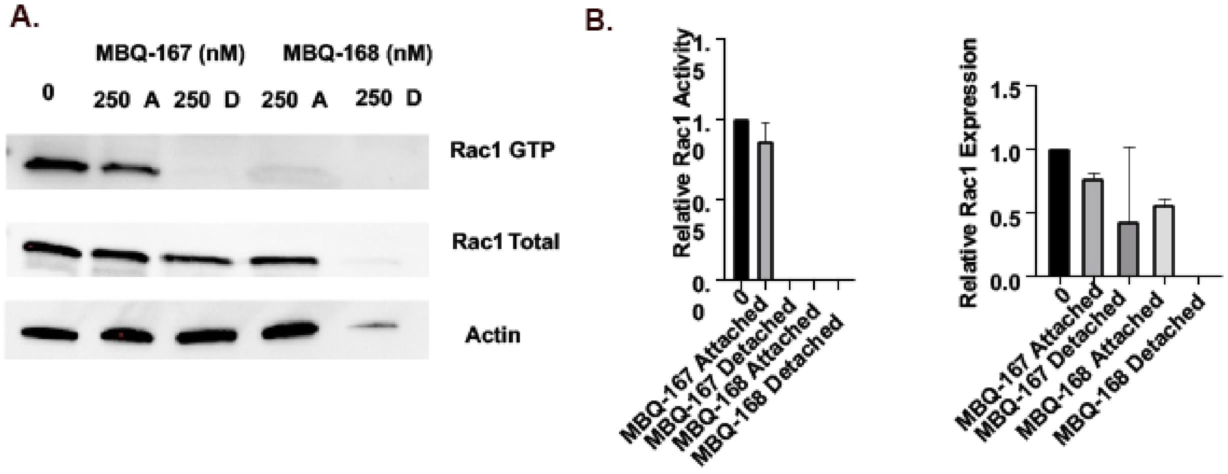

Supplementary Figure S5. **Effect of MBQ compounds on Rac activation in HER2 ++ cancer.** HER2-GFP-BM human breast cancer cells were treated for 24 hours with 250 nM of MBQ-167 and MBQ-168. Attached and detached population cells were lysed and equal amounts of proteins were subjected to pull-down assays using the p21-binding domain of PAK to isolate the GTP bound Rac1. Representative images are a result of three biological replicates.
