# Supplementary material for: Characterization of Novel Derivatives of MBQ-167, an Inhibitor of the GTP-binding Proteins Rac/Cdc42
Source: Cancer Res Commun. 2022 Dec 29;2(12):1711–26. doi: 10.1158/2767-9764.CRC-22-0303 (PMC9970268; doi:10.1158/2767-9764.CRC-22-0303)
Supplement: Suppl. Fig. S6 — Supplementary Figure S6 shows the inhibition of Rac1B, Rac1, and Rac1 P29S by MBQ-167 [file crc-22-0303-s07.pdf]

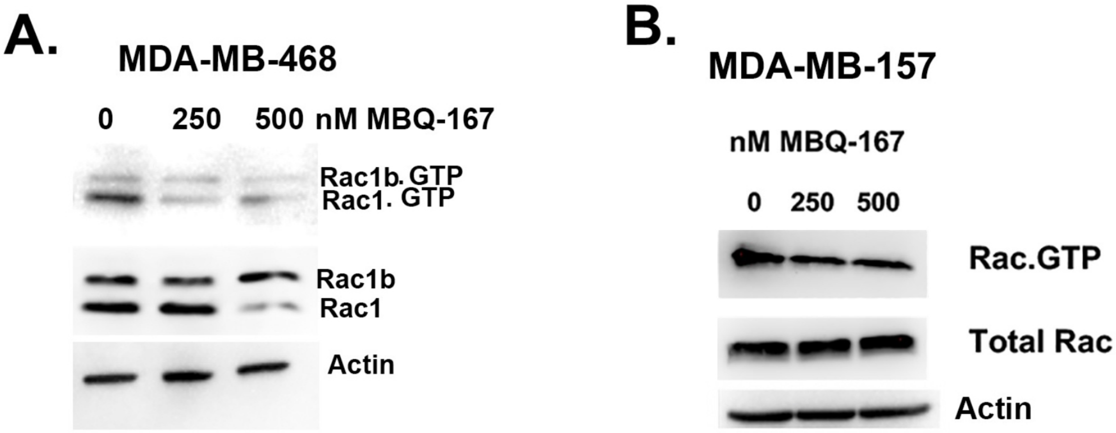

Supplementary Figure S6. MBQ-167 inhibits activation of oncogenic Rac1 in breast cancer. Effect of MBQ-167 on wild type and oncogenic Rac1 activation. **A,** MDA-MB-468 (Rac1 at 20kD and Rac1B splice variant at 23kD), and **B.** MDA-MB-157 (Rac1 P29S) TNBC cells following a pulldown assay selected for active Rac.GTP. Representative western blots immunostained for Rac1 (pulldown (GTP) or total lysate) or actin are shown.
