# Supplementary material for: Characterization of Novel Derivatives of MBQ-167, an Inhibitor of the GTP-binding Proteins Rac/Cdc42
Source: Cancer Res Commun. 2022 Dec 29;2(12):1711–26. doi: 10.1158/2767-9764.CRC-22-0303 (PMC9970268; doi:10.1158/2767-9764.CRC-22-0303)
Supplement: Suppl. Fig. S7 — Supplementary Figure S7 shows the effect of MBQ-167 on Rac1(G15A) association with Tiam-1 and p-REX-1. [file crc-22-0303-s08.pdf]

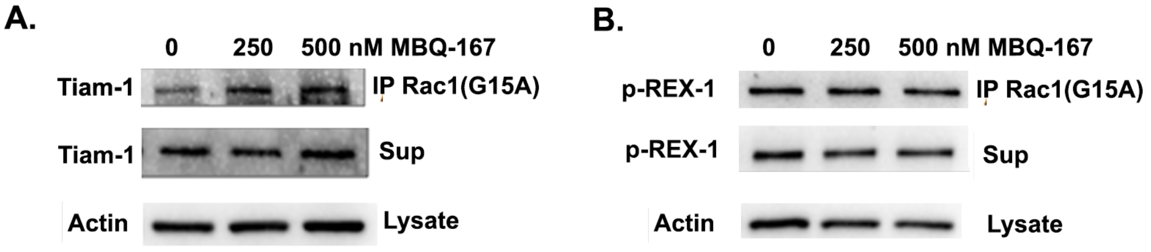

Supplementary Figure S7. **Effect of MBQ-167 on Rac association with Tiam-1or p-REX-1.** Rac1 (G15A) coupled to Glutathione sepharose beads were incubated with vehicle, 8 µM of 250 or 500 nM of MBQ-167 for 1 hr and incubated with HER2-BM cell lysates. **A**, Representative Western blots probed with Tiam-1 are shown for the Rac1 G15A pulldowns, Supernatants, and actin in the total input. **B**, Representative Western blots probed with p-REX-1 are shown for the Rac1 G15A pulldowns, Supernatants, and actin in the total input.
