# Supplementary material for: Characterization of Novel Derivatives of MBQ-167, an Inhibitor of the GTP-binding Proteins Rac/Cdc42
Source: Cancer Res Commun. 2022 Dec 29;2(12):1711–26. doi: 10.1158/2767-9764.CRC-22-0303 (PMC9970268; doi:10.1158/2767-9764.CRC-22-0303)
Supplement: Suppl. Fig. S8 — Supplementary Figure S8 shows nucleotide loading of GTP binding to Rho GTPases. [file crc-22-0303-s09.pdf]

A.

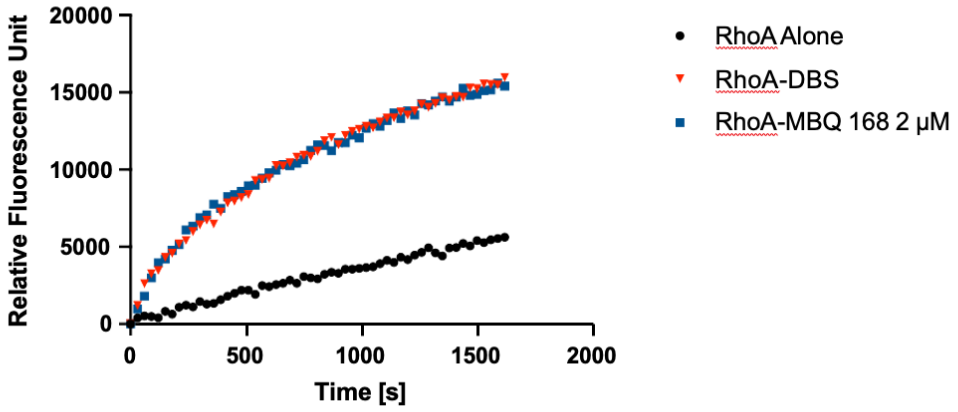

B.

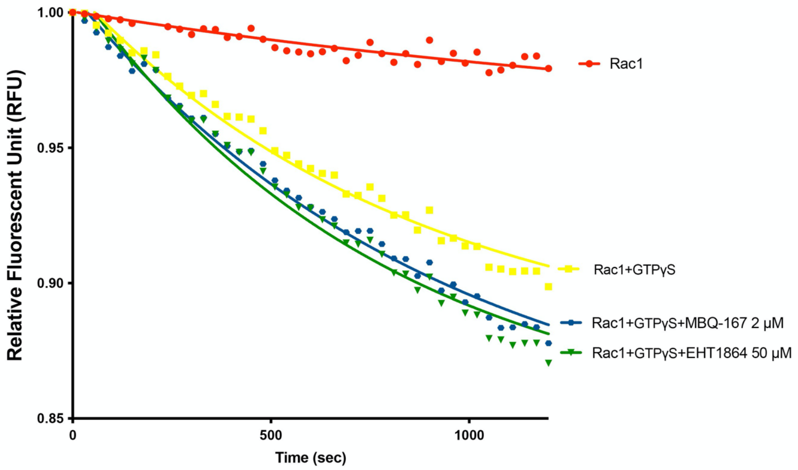

Supplemental Figure S8. Nucleotide loading of GTP binding to Rho GTPases. **A**. RhoA was incubated with 0 or 2 μM MBQ-168 and the fluorescence of N-MAR-GTP was measured. After initial five readings, the exchange factor Dbs was added to catalyze the loading of N-MAR-GTP to RhoA and monitored for 25min. **B**. 2μM Rac1 was incubated with 0 or 2 μM MBQ-167, or 50μM EHT1864 and the loss in fluorescence of Rac1 bound Bodipy-GDP was

measured when $GTP\gamma S$ was added.
